# Accumulation of ambient phosphate into the periplasm of marine bacteria is proton motive force dependent

**DOI:** 10.1038/s41467-020-16428-w

**Published:** 2020-05-26

**Authors:** Nina A. Kamennaya, Kalotina Geraki, David J. Scanlan, Mikhail V. Zubkov

**Affiliations:** 1 National Oceanography Centre, Southampton, SO14 3ZH UK; 2 School of Life Sciences, University of Warwick, Gibbet Hill, Coventry, CV4 7AL UK; 3 Diamond Light Source Ltd, Harwell Science and Innovation Campus, Chilton, Didcot, OX11 0DE UK; 4 Scottish Association for Marine Science, Scottish Marine Institute, Oban, Argyll, PA37 1QA UK; 5 Present Address: School of Plant Sciences and Food Security, The George S. Wise Faculty of Life Sciences, Tel Aviv University, Tel Aviv, 6997801 Israel

**Keywords:** Cell biology, Ecology, Microbiology, Biogeochemistry, Ecology

## Abstract

Bacteria acquire phosphate (P_i_) by maintaining a periplasmic concentration below environmental levels. We recently described an extracellular P_i_ buffer which appears to counteract the gradient required for P_i_ diffusion. Here, we demonstrate that various treatments to outer membrane (OM) constituents do not affect the buffered P_i_ because bacteria accumulate P_i_ in the periplasm, from which it can be removed hypo-osmotically. The periplasmic P_i_ can be gradually imported into the cytoplasm by ATP-powered transport, however, the proton motive force (PMF) is not required to keep P_i_ in the periplasm. In contrast, the accumulation of P_i_ into the periplasm across the OM is PMF-dependent and can be enhanced by light energy. Because the conventional mechanism of P_i_-specific transport cannot explain P_i_ accumulation in the periplasm we propose that periplasmic P_i_ anions pair with chemiosmotic cations of the PMF and millions of accumulated P_i_ pairs could influence the periplasmic osmolarity of marine bacteria.

## Abstract (web summary)

The ubiquitous oceanic bacteria harbour an external phosphate buffer for modulating phosphate (P_i_) uptake. Here, using both oceanic SAR11, *Prochlorococcus* and *Synechococcus* strains as a model, the authors show that the P_i_ buffer accumulation in the periplasm is proton motive force-dependent and can be enhanced by light energy.

## Introduction

**Fig. 1:**
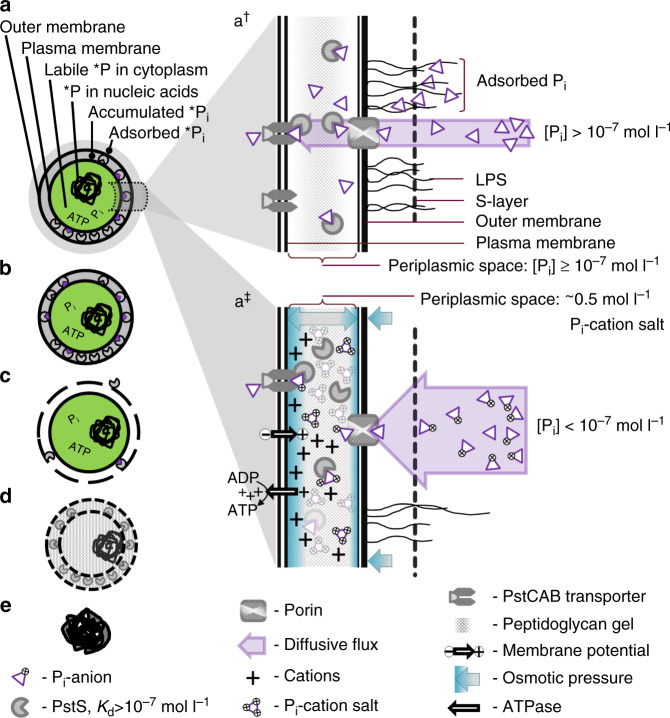
Phosphate (P_i_) acquisition by marine bacteria. **a** In a model cell labelled with *P_i_ the *P_i_-tracer can be (i) extracellular, i.e., adsorbed onto the cell surface; (ii) accumulated in the periplasm; (iii) bound to the PstS subunit in the periplasm; (iv) in a labile form in the cytoplasm, e.g., soluble P_i_, nucleotides, sugar phosphates, small molecules of RNA; and (v) in a non-labile form of assimilated P, e.g., DNA, ribosomal RNA, polyphosphates, phospholipids. **a†** Current understanding—parallel processes of passive adsorption of P_i_ to the bacterial cell surface composed of polymers of the outer membrane lipopolysaccharide (LPS) and overlaid (in some bacteria) by the proteinaceous surface layer (S-layer) and P_i_ diffusion across the outer membrane through porins into the periplasm. Diffusion is coupled directly to P_i_ transport across the cytoplasmic membrane by ABC-type transporters (PstCAB) using the periplasmic P_i_-binding protein (PstS). **a‡** The proposed model—mass transfer of P_i_ anions across the outer membrane through porins, their buffering by cations of the membrane potential in the periplasm and import of P_i_ from the buffered stock through the cytoplasmic membrane by PstCAB using P_i_-PstS. Isotonic or slightly hypertonic osmotic pressure is maintained in the periplasm by the buffered P_i_ salt. **b**–**e** Removal of different phosphorus pools by washing or fixation of a model *P_i_-labelled *Synechococcus* cell. **b** Treatment with surfactants, hydrolytic enzymes and amended ASW removes the extracellular P_i_ adsorbed to cell surface constituents. **c** A short wash with hypotonic solution, e.g., deionized water (DW), dissolves the extracellularly adsorbed P_i_ and, by causing osmotic shock, releases the periplasmic contents of a cell, i.e., the accumulated and PstS-bound P_i_. **d** Fixation of cells with paraformaldehyde (PFA) compromises the outer and inner membranes, releasing labile periplasmic and intracellular P_i_, but crosslinks cellular proteins, immobilizing the primary phosphorus-containing macromolecules including DNA, rRNA and P_i_-carrying PstS subunits. **e** Fixation of cells with trichloroacetic acid (TCA) disrupts membranes and precipitates cellular macromolecules (proteins, nucleic acids, polyphosphates and polysaccharides), thereby releasing labile P_i_ but immobilizing most of the assimilated P_i_.

Phosphorus (P) is a macronutrient universally required by all organisms including bacteria. Bacteria have a number of ways to acquire P. Scavenging P from organic molecules and reducing growth dependence on P by substituting P with sulfur in their membranes^1–6^ are both beneficial for bacteria living in phosphate (P_i_)-depleted marine environments. However, there is compelling evidence that bacteria prefer P_i_ to organic P molecules^7–9^. Marine bacteria, which typically have two membranes (and therefore stain Gram negative), acquire P_i_ using a diffusion gradient between the environment and the periplasm^10,11^. In their cells, the periplasm is separated from the environment by the outer membrane (OM), whose permeability to small, hydrophilic solutes is controlled mainly by hydrated channels—porins (Fig. 1a†). Porins select solutes on the basis of their size, shape and charge^12,13^. When nutrients such as P_i_ become depleted, cells increase the number of porins to facilitate nutrient diffusion^14^.

There are the three known bacterial transport systems to import P_i_ from the periplasm into the cytoplasm: a low affinity-high velocity phosphate inorganic transport (Pit) system, a low affinity-high velocity Na-dependent phosphate transport (Npt) system and a high affinity-low velocity phosphate-specific transport (Pst) system^15–17^. Bacteria living in P_i_-depleted waters use only the Pst system^18,19^. The PstCAB transporter is ATP-powered and receives P_i_ from a carrier protein, a PstS subunit, when the latter docks at its periplasmic side (Fig. 1a†). Although the P_i_ concentration of ~10^−7^ mol l^−1^ required for efficient import of P_i_ by PstSCAB^20^ is relatively high, it should not restrict P_i_ diffusion into the periplasm, because the periplasmic volume of even a relatively large bacterial cell, e.g., *Synechococcus* cyanobacteria (Supplementary Table 1) with an estimated periplasmic depth^21^ of 10^−8^ m, is only about 2 × 10^−17^ l. In such a tiny volume, the presence of only a few free P_i_ molecules would exceed the threshold 10^−7^ mol l^−1^ P_i_ concentration (Fig. 1a†).

Bacteria maximize the diffusive flux of nutrients through the OM by maintaining a steep nutrient concentration gradient between the environment and periplasm^10,11^. Consequently, to allow efficient diffusion of P_i_ into the periplasm in P_i_-depleted (10^−9^–10^−8^ mol l^−1^) oceanic surface waters^7,22^, the periplasmic P_i_ concentration should hypothetically be <10^−9^ mol l^−1^. This means that there should be no free P_i_ molecules in the periplasm, i.e., every P_i_ molecule entering the periplasm should be instantly bound by a PstS subunit requiring an affinity >100 times above the known PstS affinity limit. However, the PstS affinity requirement does not seem to limit the growth of ubiquitous SAR11 alphaproteobacteria and *Prochlorococcus* cyanobacteria—the two bacterial populations comprising ≤¾ of oceanic surface bacterioplankton in the P_i_-depleted North Atlantic subtropical gyre^7^. Furthermore, the ecological success of these bacteria is probably related to the high rates of P_i_ uptake measured in the gyre^7,22^. Surprisingly, measured rates of P_i_ acquisition by *Prochlorococcus* and SAR11 are lower in tropical surface waters where bacterial growth and P_i_ concentrations are higher^22^. The counter-intuitive reduction in the P_i_ acquisition rate by faster growing bacteria was attributed to the presence of an intermediate buffer, in which both SAR11 and *Prochlorococcus* cells store P_i_: the fuller the P_i_ buffer is, e.g., in P_i_-replete tropical surface waters, the fewer P_i_ molecules a cell acquires slower from seawater to top the buffer up^22^. As every P_i_ molecule acquired, or more precisely accumulated, by a cell first enters the buffer before being imported for assimilation, bacterial P_i_ uptake into the buffer and P_i_ import from the buffer can be decoupled. Although *Prochlorococcus* and SAR11 bacteria possess genes related to synthesis of polyphosphates^23^, their cells are perhaps too small to store P_i_ as polyphosphates intracellularly and they somehow accumulate P_i_ extracellularly.

Our aim here is to explain how marine bacteria accumulate P_i_ extracellularly. We demonstrate that P_i_ accumulation rates by oceanic SAR11, *Prochlorococcus* and *Synechococcus* cells are extremely variable in the P_i_-depleted North Atlantic. Lower rates can be stimulated by light energy but their maximal rates are insensitive to light and approach the theoretical upper limit of diffusion. To relate the accumulated P_i_ with cellular P_i_ requirements, the P contents of flow-sorted cells of oceanic *Prochlorococcus* and SAR11 were determined by microbeam synchrotron X-ray fluorescence (μ-SXRF) using flow-sorted cultured *Synechococcus* cells for calibration. Using pulse-chase experiments with ^33^P_i_ and ^32^P_i_ (*P_i_) radiotracers, we showed that cultured *Synechococcus* cells accumulate P_i_ in a similar way to SAR11 and *Prochlorococcus* cells^22^. This justified the use of *Synechococcus* isolates for more intrusive tests (e.g., of retention of accumulated P_i_), to curtail artefacts resulting from the low tolerance of oceanic SAR11 and *Prochlorococcus* bacteria to harsh experimental manipulations. Moreover, by working with artificial seawater (ASW), we expanded the experimental range of P_i_ concentrations below those encountered in natural P_i_-depleted seawater. To examine how the extracellular P_i_ is retained, we developed a living cellular model system with the extracellular *P_i_-labelled buffer (Fig. 1a) and treated them with hydrolytic enzymes, surfactants, inhibitors, amended ASW and hypotonic solutions to locate whether the P_i_ buffer is on the cell surface (Fig. 1b), e.g., retained by a charged S-layer, as was recently proposed for archaea^24^, or in the periplasm (Fig. 1c). We used specific metabolic inhibitors^25–28^ to determine whether the proton motive force (PMF) is driving extracellular P_i_ accumulation and retention.

Based on our field and laboratory experiments, we conclude that the PMF is essential for P_i_ accumulation (but not retention) in the periplasm of marine bacteria. As the PstS-driven import cannot explain P_i_ accumulation in the periplasm, we propose a mechanism of periplasmic ionic pairing of inwardly diffusing P_i_ anions with chemiosmotic cations of the membrane potential (Fig. 1a‡), which could conserve cellular energy and control periplasmic turgor.

## Results

### Bacterial clearance rates of P_i_

**Fig. 2:**
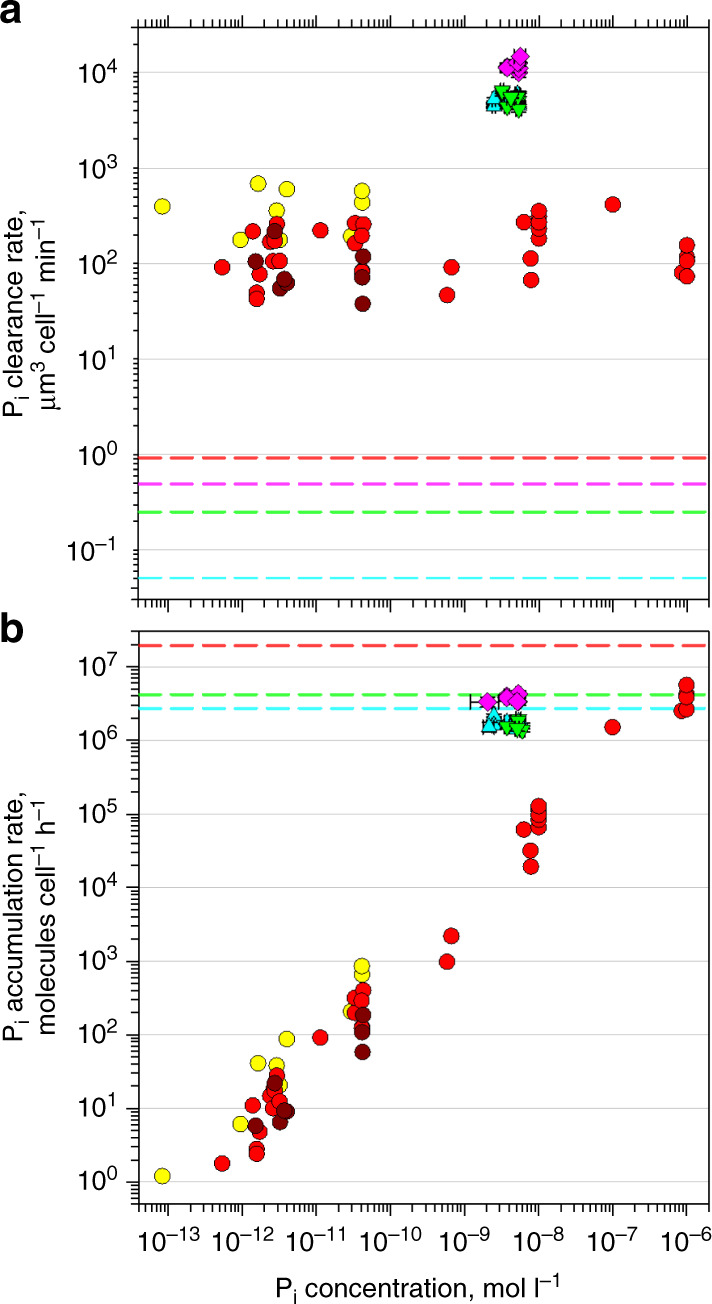
P_i_ acquisition rates of oceanic and cultured cells. **a** Comparison of cellular P_i_ clearance rates of oceanic SAR11 (light blue triangles, *n* = 14), *Prochlorococcus* (green triangles, *n* = 11) and *Synechococcus* (pink diamonds, *n* = 7) at ambient P_i_ concentrations with rates of cultured *Synechococcus* strains WH7803 (yellow circles, *n* = 10), WH8102 (red circles, *n* = 35) and WH8109 (brown circles, *n* = 8) across a wide range of P_i_ concentrations. **b** Comparison of maximal cellular P_i_ accumulation rates of the same bacteria (SAR11 *n* = 12, *Prochlorococcus* *n* = 7, *Synechococcu* *n* = 7, WH7803 *n* = 10, WH8102 *n* = 35 and WH8109 *n* = 8). Dashed lines indicate corresponding (**a**) cellular volumes (µm^3^ cell^−1^) and (**b**) cellular phosphorus content (P atoms cell^−1^) of oceanic SAR11 (light blue), *Prochlorococcus* (green), *Synechococcus* (pink) and cultured *Synechococcus* sp. WH8102 (orange). Horizontal grey lines indicate major ticks of the logarithmic Y-axis to assist comparing values. Symbols present individual measurements for cultured *Synechococcus* cells and mean values of four sorting replicates for oceanic bacteria; error bars indicate corresponding SD. The full range of rates of oceanic bacteria is shown in Supplementary Fig. 2. Source data are provided as a Source Data file.

P_i_ clearance rates (the volume of water cleared of P_i_ by a cell per unit of time, while the cell accumulates the cleared P_i_) of oceanic SAR11, *Prochlorococcus* and *Synechococcus* cells vary by orders of magnitude in the patchily P_i_-depleted North Atlantic (Supplementary Figs. 1 and 2a, and Supplementary Table 2). Living in such a patchy environment warrants the use of an extracellular buffer, where P_i_ is temporarily stored^22^. Mostly the measured rates are lower in waters adjacent to the subtropical gyre with higher in situ P_i_ concentrations, whereas the rates are higher in the central gyre waters with lower P_i_ concentrations (18–28°N, Supplementary Fig. 2a and Supplementary Table 2). In the central gyre waters, maximal P_i_ clearance rates of SAR11, *Prochlorococcus* and *Synechococcus* cells (Fig. 2a) are only 30×, 40× and 25×, respectively, below the theoretical maximal rate of nutrient acquisition by diffusion^10,11^. Apparently, a *Synechococcus* cell can accumulate P_i_ faster than either a *Prochlorococcus* or a SAR11 cell. To understand why the abundance of *Synechococcus* in the central gyre waters is three orders of magnitude lower than the abundance of *Prochlorococcus* and SAR11, we need to take into account cell sizes and cellular P requirements of these bacteria.

Once their maximal P_i_ clearance rates are normalized to cellular biovolumes (Supplementary Table 1 and Fig. 2a) then a *Prochlorococcus*, a *Synechococcus* or a SAR11 cell can theoretically clear all dissolved P_i_ from seawater ≤2.5 × 10^4^, 2.6 × 10^4^ or 1.2 × 10^5^ times its cellular volume min^−1^, respectively. In agreement with the surface area-to-volume concept^29^, these biovolume-specific clearance rates show that a SAR11 cell is noticeably (4–5×) more effective in uptake of P_i_ than either a *Prochlorococcus* cell or a *Synechococcus* cell probably owing to the 2× higher surface area-to-volume ratio of a small, curved-rod shape SAR11 cell compared with the cocci cyanobacteria (Supplementary Fig. 3 and Supplementary Table 1).

To account for the cellular Ps requirements of oceanic bacterial cells, we determined their cellular P content. The cellular P content of a *Synechococcus* cell (1.93  × 10^7^ P atoms cell^−1^) and hence their P demand is 5–7× higher than the P content of a *Prochlorococcus* and a SAR11 cell (4.14 and 2.68 × 10^6^ P atoms cell^−1^, respectively, Fig. 2b and Supplementary Table 3). This implies that *Synechococcus* growth in P_i_-depleted waters is constrained by its high P demand rather than by its ability to acquire P_i_. It is also notable that a larger *Synechococcus* and a smaller *Prochlorococcus* have similar biovolume-specific P_i_ clearance rates, despite the 1.2× higher cell surface-to-volume ratio of the latter (Supplementary Table 1). As porin-restricted diffusion constrains the P_i_ clearance rate, the OM of *Synechococcus* ought to be more permeable to P_i_, e.g., to have more P_i_-specific porins per µm^2^ of the OM surface.

The above comparisons show that despite its high P demand, a *Synechococcus* cell is a good model system for studying the process of P_i_ acquisition by oceanic bacteria. In our laboratory studies, we focused on the oligotrophic *Synechococcus* sp. strain WH8102 but also made comparisons with the mesotrophic *Synechococcus* strains WH8109 and WH7803^30^. The 40–90× lower P_i_ clearance rate of larger cells of cultured *Synechococcus* compared with oceanic bacterioplankton suggests that *Synechococcus* cells grown at a P_i_ concentration >10^−5^ mol l^−1^ have fewer porins^12,13^ than oceanic bacteria even when the P_i_ concentration is downshifted (Fig. 2a). Such porin-restricted diffusion explains why a cultured *Synechococcus* cell cleared P_i_ from about the same volume of water, while the P_i_ concentration ranged over more than seven orders of magnitude. The P_i_ clearance rate of cultured *Synechococcus* cells remained relatively constant between the three strains down to the low end of the tested P_i_ concentrations (Fig. 2a), such that within a few hours these cells removed virtually all ^33^P_i_ or ^32^P_i_ tracers from the medium (Supplementary Table 4). Using carrier-free ^32^P_i_ tracer, we determined that the final P_i_ concentration in the surrounding seawater was depleted by *Synechococcus* to 10^−15^ mol l^−1^ (the instrument detection limit), or six orders of magnitude below oceanic P_i_ levels (Supplementary Table 2).

### Fast growth-decoupled accumulation of P_i_

The rate of P_i_ accumulation (the amount of P_i_ molecules accumulated by a cell per unit of time) by cultured *Synechococcus* cells was proportional to the P_i_ concentration over a range of concentrations from 10^−13^ to 10^−6^ mol l^−1^ (Fig. 2b), much wider than the range of 10^−9^–10^−8^ mol l^−1^ offered by the P_i_-depleted North Atlantic (Supplementary Table 2). At P_i_ concentrations similar to P_i_-depleted oceanic waters, cultured cells accumulated P_i_ orders of magnitude slower than oceanic SAR11, *Prochlorococcus* and *Synechococcus* cells (Fig. 2b and Supplementary Fig. 2b). At 10^−6^ mol P_i_ l^−1^, a cultured *Synechococcus* cell can, however, double its cellular P content within merely 3 h (Fig. 2b and Supplementary Table 3), while they divide once every 1–2 days. Similarly, when related to their cellular P content, oceanic *Prochlorococcus* and SAR11 cells obtained sufficient P_i_ for cell division in merely 1–2 h, whereas in such oligotrophic waters *Prochlorococcus* divide only once every 3.5 days^31^ and SAR11 once every 2–3 days (calculated based on their cellular leucine uptake rates^32^). This decoupling of P_i_ accumulation and growth rates in *Synechococcus*, *Prochlorococcus* and SAR11 cells suggests that, apart from being a reserve for steady growth, the accumulated P_i_ could have an additional, important cellular function.

### *Synechococcus* cells accumulate P_i_ extracellularly

**Fig. 3:**
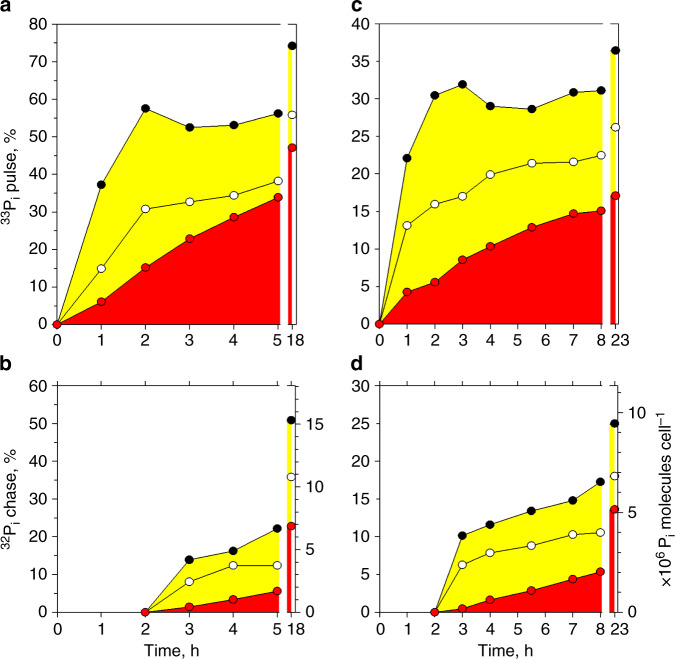
P_i_ accumulation by *Synechococcus* sp. WH8102. Left (**a**, **b**) and right (**c**, **d**) panels show the results of two representative pulse-chase experiments with cells at exponential and early stationary growth phases, respectively. **a**, **c** Accumulation dynamics of 10^−8^ mol l^−1 33^P_i_-pulse added at 0 h in live cells washed with artificial seawater − the total P_i_ (black circles), live cells washed with deionized water − the cytoplasmic P_i_ (white circles) and in the PFA-fixed cellular biomass − the macromolecule-bound P_i_ (red circles), as a percentage of added tracer. **b**, **d** Accumulation dynamics of the 10^−6^ mol l^−1 32^P_i_-chase added at 2 h as the total P_i_, the cytoplasmic P_i_ and the macromolecule-bound P_i_. The additional right Y-axis (**b**, **d**) shows cellular rates of ^32^P_i_-chase accumulation. To assist comparison of the labile and non-labile P_i_, the areas below lines are coloured yellow for the labile P_i_ and red for non-labile P_i_. Symbols are connected with lines to indicate the slopes of the ^33^P_i_ and ^32^P_i_ tracer accumulation. The X-axis is broken to add an additional, final time point. Circles present mean values of technical duplicates or individual measurements. Source data are provided as a Source Data file.

To assess whether cultured *Synechococcus* cells accumulate P_i_ in the extracellular buffer, the accumulation of the ^33^P_i_-pulse and ^32^P_i_-chase was determined in live WH8102 cells (total P_i_, Fig. 1a) and in their cellular macromolecules (biomass fixed with paraformaldehyde (PFA) that immobilizes DNA and RNA—the two principal P_i_-containing macromolecules—as well as some periplasmic constituents, including PstS subunits) (Fig. 1d). After addition of the 10^−6^ mol l^−1 32^P_i_-chase, accumulation of the ^33^P_i_-pulse into cellular macromolecules should be halted, because any of the 4.5–7 × 10^−9^ mol l^−1 33^P_i_-pulse that remains in the seawater would be diluted with the chase >100×. However, similar to that observed in oceanic bacteria^22^, the pulsed ^33^P_i_ continued to be assimilated into cell macromolecules at an unchanged rate, which started slowing down only after 5 h (Fig. 3a, c). Usually in pulse-chase experiments, incorporation of a pulsed molecule (e.g., amino acid or nucleoside) into bacterial macromolecules halts abruptly upon dilution with the chase, because the intermediate cellular labile pool of the pulsed molecule is small, e.g., 5% of the tracer incorporated into macromolecules^33^. In the case of P_i_, that labile pool was 10×–20× larger than the amount assimilated into cellular macromolecules (Fig. 3b, d at the 3 h time point).

**Fig. 4:**
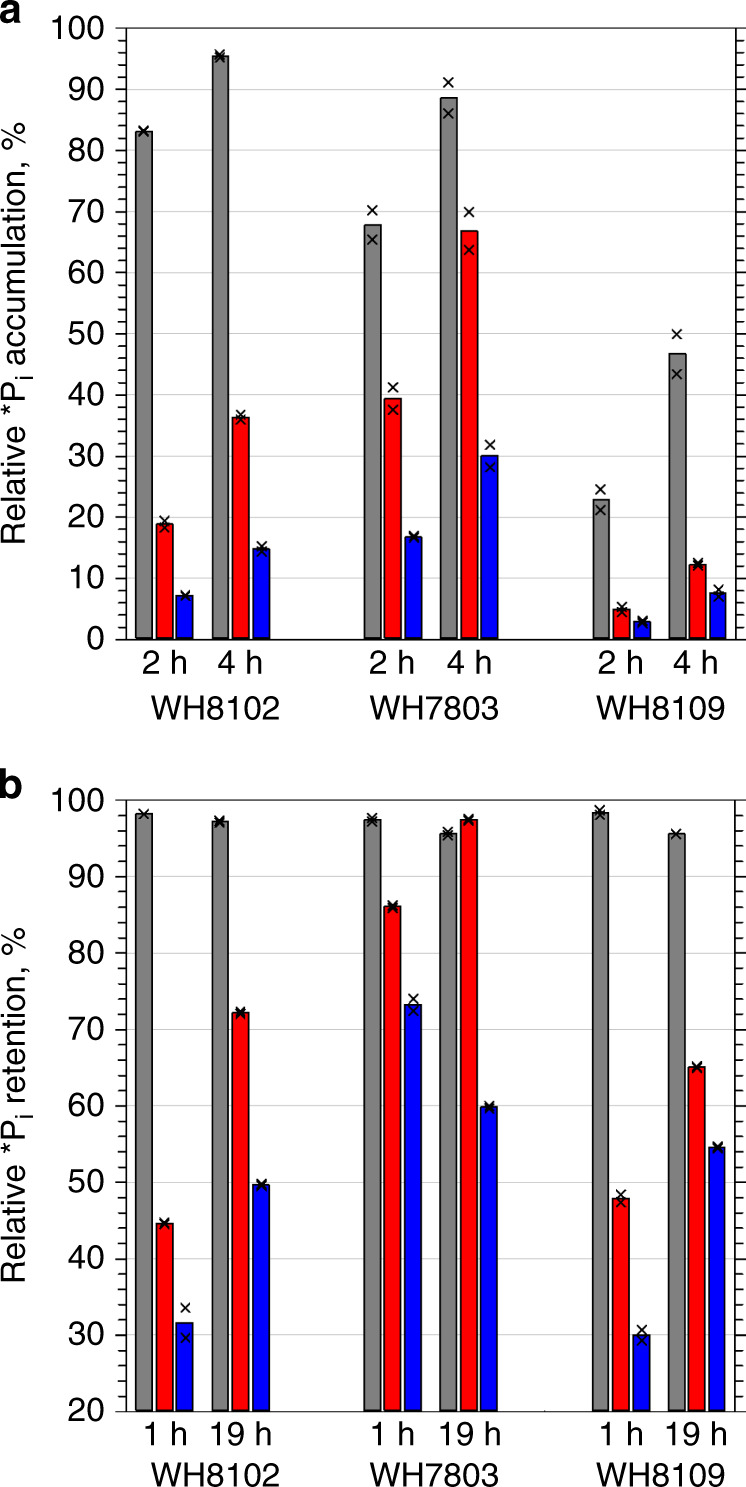
Accumulation and retention of the accumulated *P_i_ by *Synechococcus*. **a** The total P_i_ accumulated in live cells (grey bars) compared with the amount of P_i_ in PFA-fixed cellular macromolecules (red bars) and TCA-precipitated nucleic acids (blue bars). Cells washed to remove the free P_i_ were re-suspended in ASW-P_i_, supplemented with 100 Bq ml^−1^ of H_3_^32^PO_4_, incubated under standard cultivation conditions and sampled after 2 and 4 h. **b** Retention of the total accumulated P_i_ in *P_i_ pre-labelled cells and its internalization into macromolecules and nucleic acids. The pre-labelled cells re-suspended in ASW-P_i_ were incubated under standard cultivation conditions and sampled after 1 and 19 h. The plots show the results of representative experiments. Bars present mean values of technical duplicates indicated as crosses. Horizontal grey lines indicate major ticks of the Y-axis to assist comparing values. Source data are provided as a Source Data file.

We then used the difference in *P_i_ label between the total P_i_ in live cells and macromolecule-bound P_i_ to estimate the amount of labile P_i_ in three *Synechococcus* strains. This showed the total *P_i_ content of tested live cells (Fig. 4a) to be consistently several times higher than the *P_i_ content of cellular macromolecules (fixed with either PFA or trichloroacetic acid (TCA), Fig. 1d,e and Supplementary Fig. 4). Furthermore, in the *P_i_ pre-labelled cells, which were washed and suspended in ASW with no *P_i_ (see Methods), the *P_i_ content in macromolecules increased with time and approached the total *P_i_ that remained stable (Fig. 4b). With no *P_i_ in the medium, the additional *P_i_ gained by the macromolecules can only be sourced from *P_i_ accumulated in the intermediate buffer. These data also show that the accumulated *P_i_ is semi-labile and can be imported into the cytoplasm to synthesize nucleic acids.

**Fig. 5:**
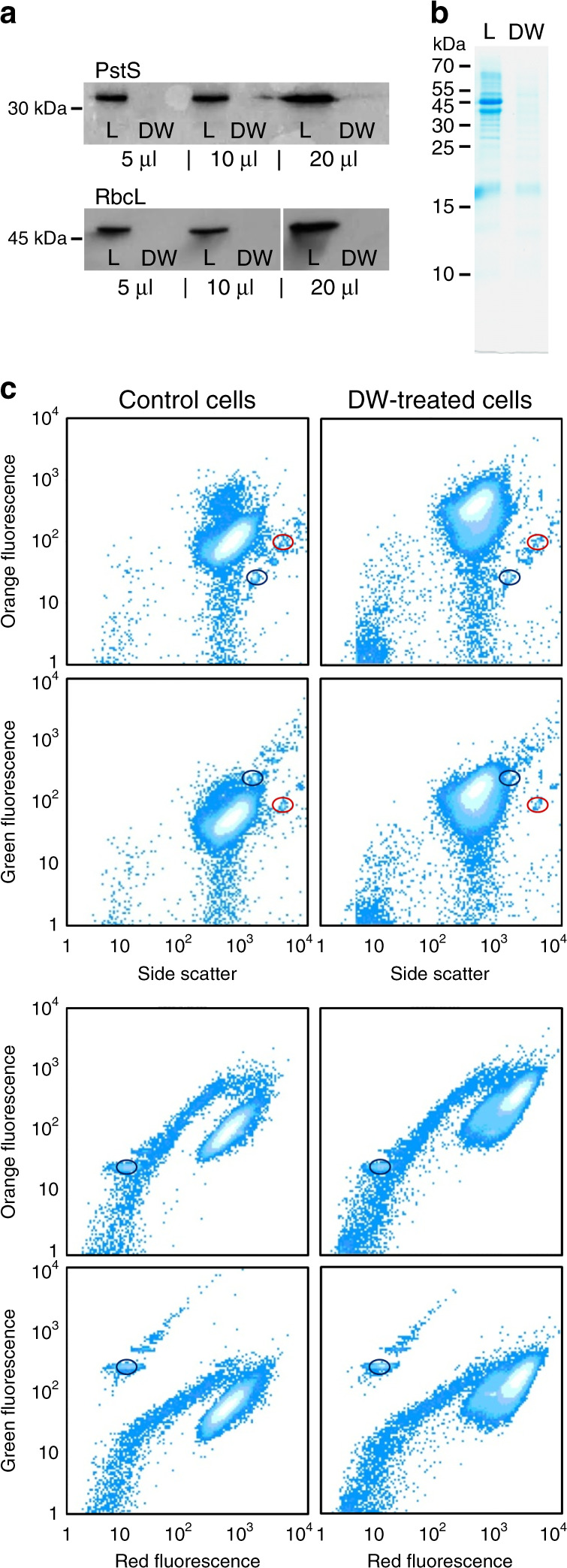
Hypotonic release of the periplasm without lysing *Synechococcus* sp. WH8102 cells. Western blotting of periplasmic and cell lysate proteins (**a**, **b**). Cells were subjected to 1 min hypotonic shock in deionized water (DW) and pelleted to separate treated cells from supernatant. Proteins present in the DW supernatant were compared with proteins in the lysate (L) of treated cells. **a** Western blot immunolabeling with antibodies against the periplasmic P_i_-binding subunit PstS and the large Rubisco subunit RbcL (5, 10 or 20 μl per lane) reveals the presence of PstS but no signal for RbcL in the DW supernatant. **b** Coomassie-stained SDS-PAGE (10 μl per lane) with the size range of proteins in the L and DW samples is shown for reference. Mw, molecular-weight size marker (kDa). The experiment was repeated independently four times with similar results. Comparison of control (left column) and DW-treated cells (right column) using flow cytometric pseudo-colour plots (**c**) of cellular orange or green autofluorescence vs. red autofluorescence or side scatter. Visualized in BD CellQuest™ Pro, cellular light scatter and autofluorescence of photosynthetic pigments cluster cells in a single cytometric population separate from a dispersed cluster of other suspended particles and tight clusters of reference beads. Compared with the control cells incubated in ASW medium, the cells incubated in DW for 0.5 h have higher autofluorescence but still form a single population, which shows minor deterioration, i.e., cell lysis. Cells were analysed without fixation or staining. The yellow-green (505/515 nm) 0.5 μm beads (circled in dark blue) and multifluorescence 1.0 μm beads (circled in red) were used as an internal reference. Source data are provided as a Source Data file.

To confirm the extracellular (i.e., not in the cytoplasm) location of the labile P_i_, we washed cells for 1–2 min with deionized water (DW). Washing live cells with DW effectively strips P_i_ and other ions adsorbed to the OM components. In addition, such brief hypo-osmotic shock also releases the content of the periplasm without disrupting the plasma membrane. Western blotting analysis confirmed the presence of the periplasmic P_i_-binding subunits PstS in the DW-washed fraction but detected no Rubisco, arguably the most abundant soluble protein in the cyanobacterial cytoplasm (Fig. 5a, b). In fact, even a 30 min incubation of *Synechococcus* in DW did not lyse the cells (Fig. 5c). As a substantial fraction of the accumulated *P_i_ was removed with a brief rinse of live *Synechococcus* with DW (Fig. 3), we therefore conclude that their cells accumulate P_i_ outside the plasma membrane, i.e., on the surface of the OM or in the periplasm (Fig. 1a†).

### Periplasmic location and retention of the P_i_ in *Synechococcus*

**Fig. 6:**
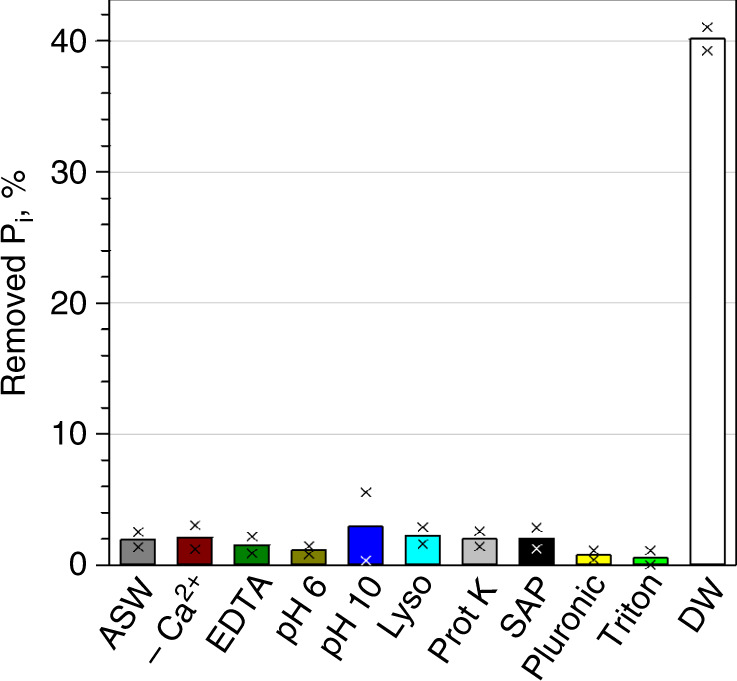
Retention of the accumulated *P_i_ by live, pre-labelled *Synechococcus* sp. WH8102 cells. Minor removal of P_i_ label was achieved by the following treatments: ASW, cells washed with artificial seawater; -Ca^2+^, cells washed with ASW without Ca^2+^; EDTA, cells washed with isotonic NaCl solution containing EDTA; pH 6 and pH 10, cells washed with ASW at pH 6 and at pH 10, respectively; Lyso and Prot K, cells incubated with lysozyme and Proteinase K, respectively for 15 min; SAP, cells incubated with shrimp alkaline phosphatase for 30 min; Pluronic and Triton, cells incubated with the surfactants Pluronic F-68 and Triton X-100, respectively, for 15 min. A short wash with deionized water (DW) removed about 40% of the P_i_ label. The plot summarizes the results of the representative experiments. Bars present mean values of technical duplicates indicated as crosses. Horizontal grey lines indicate major ticks of the Y-axis to assist comparisons. For details, refer to Supplementary Tables 5 and 6. Source data are provided as a Source Data file.

To identify the exact location of the accumulated P_i_ and to assess whether P_i_ was associated with specific cell surface ligands, we examined the response of live *P_i_ pre-labelled *Synechococcus* cells to a variety of treatments, which are inapplicable to delicate oceanic bacteria (Fig. 1b, c and Supplementary Tables 5 and 6). Treatments with surfactants released no *P_i_ (Fig. 6), suggesting that it is not associated directly with OM lipids. High concentrations of a broad-spectrum protease Proteinase K, which was shown to efficiently digest extracellular proteins in bacteria^34,35^, did not release the accumulated *P_i_, implying no direct involvement of extracellular proteins (including the S-layer proteins of WH8102^36^) in P_i_ accumulation (Fig. 6). Release of the accumulated P_i_ did not happen after destabilization of the spatial conformation of lipopolysaccharides (LPS) by depletion of Ca^2+^ and Mg^2+^ di-cations^37^ (ASW-P_i_-Ca and NaCl + EDTA) or by lysozyme treatment^38^ (Fig. 6), suggesting no direct involvement of LPS in P_i_ accumulation. Retention of P_i_ by phosphorylation of extracellular macromolecules was ruled out, because alkaline phosphatase had no effect on the accumulated P_i_ (Fig. 6). Incubations with either acidic (pH 6) or alkaline (pH 10) ASW failed to remove the accumulated P_i_ (Fig. 6), demonstrating an insignificant effect of (de)protonation on the stability of the accumulated P_i_.

**Fig. 7:**
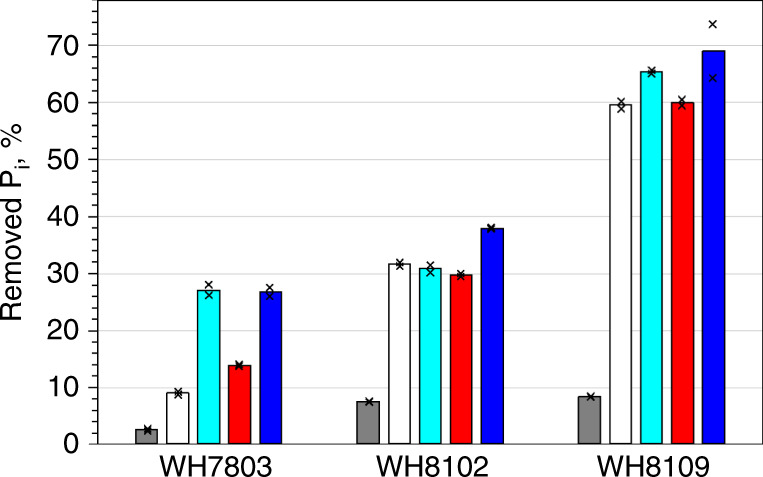
Osmotic removal of the accumulated P_i_ from the *P_i_ pre-labelled cells. Comparison of removable *P_i_ from live cells of the three *Synechococcus* strains incubated for 1 h and washed with artificial seawater (grey bars), deionized water (white bars) and phosphate-buffered saline (turquoise bars) vs. PFA-fixed cellular macromolecules (red bars) and TCA-precipitated nucleic acids (blue bars). The plot shows the results of a representative experiment. Bars present mean values of technical duplicates indicated as crosses. Horizontal grey lines indicate major ticks of the Y-axis to assist comparisons. Source data are provided as a Source Data file.

To assess the role of PMF in retention of the accumulated P_i_, we treated *P_i_ pre-labelled *Synechococcus* cells with the inhibitor carbonyl cyanide m-chloro-phenyl-hydrazone (CCCP) (Supplementary Table 7). Dissipation of the PMF had a minor effect on the already accumulated P_i_: the amount of released P_i_ was comparable to the amount of P_i_ lost by washing live cells with ASW-P_i_ (Supplementary Fig. 4). In contrast to the above treatments, accumulated P_i_ was easily removed by short (1–2 min) rinses of live cells with a range of hypotonic solutions (Fig. 7), i.e., ASW diluted 1:10–1:100, phosphate-buffered saline or DW (Supplementary Table 8), which release periplasmic cell contents^27,39^ but do not cause osmotic cell lysis of marine *Synechococcus* (Fig. 5). Hypotonic washes reproducibly removed an amount of *P_i_ less than, or similar to, that removed from the PFA-fixed cellular macromolecules (Figs. 3 and 7). Thus, resistance to a variety of extracellular treatments (Fig. 6) in conjunction with the hypotonic removal of the accumulated P_i_ (Figs. 3, 5 and 7) strongly indicates the periplasmic location of the accumulated P_i_.

### The PMF is essential for P_i_ accumulation

**Fig. 8:**
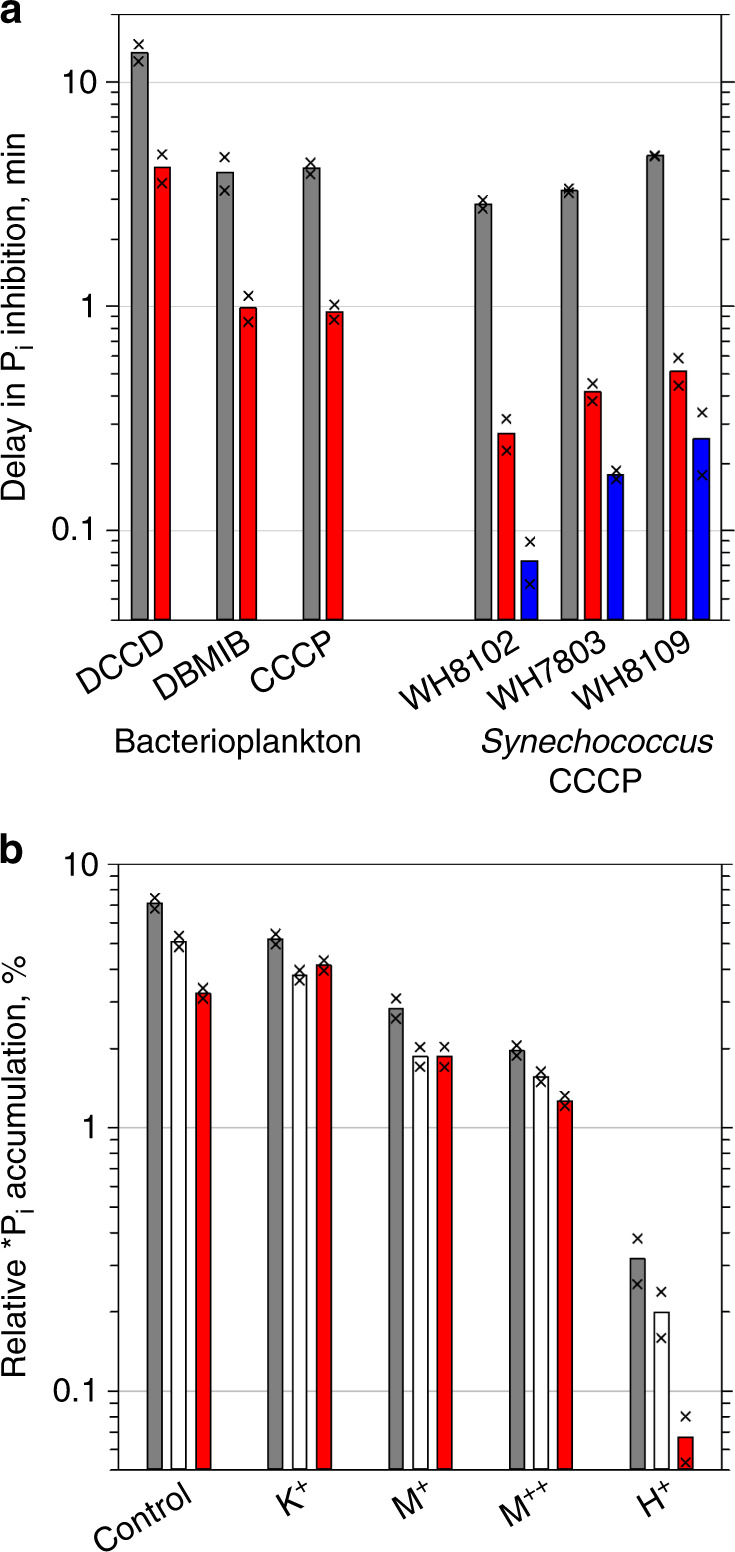
Inhibition of bacterial P_i_ accumulation. **a** Comparison of the time delay in the inhibition of *P_i_ accumulation between bacterioplankton treated with DCCD, DBMIB and CCCP, and *Synechococcus* strains WH8102, WH7803 and WH8109 treated with CCCP. The time delay is derived from the rate of P_i_ accumulation, measured for uninhibited control cells. **b** Relative P_i_ accumulation in *Synechococcus* sp. WH8102 cells treated with the K^+^-specific ionophore Valinomycin (K^+^), the monovalent metal-specific ionophore Monensin (M^+^), the divalent metal-specific ionophore A23187 (M^++^) and the protonophore CCCP (H^+^). Plots show the results of representative experiments. Bars present mean values of technical duplicates indicated as crosses. Horizontal grey lines indicate major ticks of the logarithmic Y-axis to assist comparing values for the following treatments: P_i_ accumulation into live cells washed with seawater (grey bars), into PFA-fixed cellular macromolecules (red bars) and into TCA-fixed cellular nucleic acids (blue bars) (**a**) or into the cytoplasm (white bars) of cells washed with deionized water (**b**). Source data are provided as a Source Data file.

The inhibitor CCCP has previously been used to investigate the role of the PMF in various aspects of cyanobacterial physiology^26,27^. Although we used this inhibitor to specifically elucidate the role of the PMF in P_i_ accumulation by cultured *Synechococcus* cells, we compared the CCCP effect with the effect of three other metabolic inhibitors (Supplementary Table 7) on P_i_ acquisition by oceanic bacterioplankton to safeguard from potential artefacts. A similar time course of P_i_ clearance by the control and 3-(3,4-dichloro-phenyl)-1,1-dimethylurea (DCMU)-treated bacterioplankton demonstrates that DCMU (which reduces photosynthetic electron flow and proton extrusion) slows down the microbial P_i_ clearance rate by ~30% (Supplementary Fig. 5). This reduction is consistent with the percentage (~30%) of cyanobacterial cells within the total bacterioplankton. The other three inhibitors completely terminated P_i_ clearance by bacterioplankton, as no time course was observed (Supplementary Fig. 5). The effect of N,N'-dicyclohexyl-carbodiimide (DCCD, an ATPase inhibitor) was delayed three times longer compared with 2,5-dibromo-3-methyl-6-isopropylbenzo-quinone (DBMIB) and CCCP, which halted P_i_ accumulation after 4 min (Fig. 8a, grey bars).

Likewise, CCCP was a highly effective inhibitor of P_i_ accumulation by cultured *Synechococcus* cells (Fig. 8a). The time delay of 3–5 min common to the three strains was similar to the 4 min delay of CCCP-treated bacterioplankton. This abrupt halt of P_i_ accumulation by CCCP-treated *Synechococcus* cells indicates that, as in experiments with bacterioplankton (Fig. 8a), membrane depolarization rather than ATP depletion is behind the inhibition, whereas the generic 4 min delay presumably reflects the time required for CCCP molecules to integrate into cellular membranes. As there was <1 min to incorporate *P_i_ into the PFA-fixed macromolecules and even less time to incorporate it into TCA-fixed nucleic acids (Fig. 1d, e), the bulk of the accumulated P_i_ in CCCP-treated cells (32% and 11% of the total P_i_ was in the PFA-fixed macromolecules of oceanic bacteria and isolates, respectively) remained labile (Fig. 8a). This comparison of live and fixed cells worked equally well for bacterioplankton and for cultures (Fig. 8a), confirming that in all bacteria tested P_i_ is first accumulated in the periplasm before being imported into the cytoplasm and assimilated.

The PMF consists of the transmembrane chemical H^+^ gradient (ΔpH) and the transmembrane electric gradient (ΔΨ) that can be sustained by other cations. The protonophore CCCP dissipates both the ΔpH and ΔΨ. To specifically link one of the components to P_i_ accumulation in the periplasm, we abolished ΔΨ in WH8102 using the three cation-specific ionophores: a K^+^-specific ionophore (Valinomycin), a monovalent cation-specific (e.g., Na^+^ and K^+^) ionophore (Monensin) and a divalent metal-specific (e.g., Mn^2+^, Ca^2+^ and Mg^2+^) ionophore (A23187, Supplementary Table 7), which facilitate the electroneutral transport of cations through a membrane down the chemical gradient. Compared with the control, the three ionophores reduced P_i_ accumulation into live cells by 0.4×, 1.5× and 2.6×, respectively (Fig. 8b). A significant fraction (20–37%) of the accumulated P_i_ remained in the periplasm. As relative to the protonophore CCCP, the three ionophores had a minor effect on P_i_ accumulation by *Synechococcus* cells (Fig. 8b), we conclude that ΔpH rather than ΔΨ is crucial for the periplasmic P_i_ accumulation. To validate conclusions about energy-dependent accumulation of P_i_, drawn from the inhibitor-based experiments, we tested whether P_i_ accumulation by SAR11, *Prochlorococcus* and *Synechococcus* cells could be energy-stimulated. Conveniently, these bacteria use light energy in their metabolism.

### Light stimulation of P_i_ accumulation

**Fig. 9:**
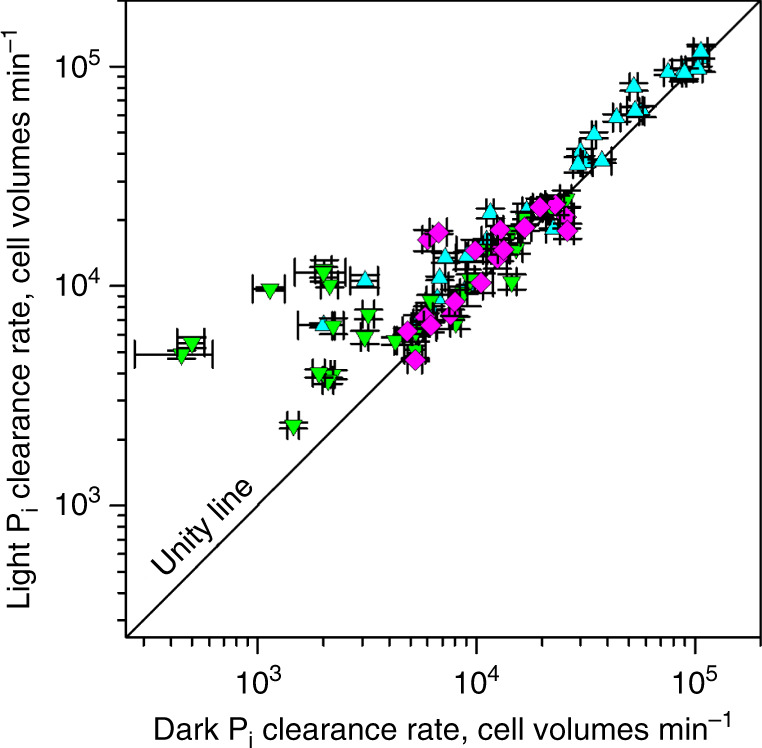
Comparison of P_i_ clearance rates of oceanic cells in the light versus dark. Comparison of P_i_ clearance rates in cell volume equivalents of oceanic SAR11 (turquoise triangles, *n* = 28), *Prochlorococcus* (green triangles, *n* = 32) and *Synechococcus* (pink diamonds, *n* = 19) at ambient P_i_ concentrations. Equivalents of cell volumes cleared of P_i_ per minute were used to negate the bacterial size differences. Symbols present mean values of four sorting replicates of the paired light vs. dark experiments; error bars indicate corresponding SD. Source data are provided as a Source Data file. Values above the unity line indicate light stimulation.

To test whether light can energize bacterial P_i_ accumulation, we compared P_i_ clearance by oceanic bacterioplankton cells incubated in the light vs. dark (Fig. 9). Indeed, light enhanced P_i_ accumulation by all cells studied (Supplementary Table 9). Compared with spectacular (≤10 times) but sporadic light stimulation of P_i_ clearance by *Prochlorococcus* cells (and to a lesser extent by *Synechococcus* cells), light stimulation of P_i_ clearance by SAR11 cells was more uniform, averaging 40% (Fig. 9). Counter-intuitively, light stimulation was distinctly more pronounced at lower rates of P_i_ clearance in moderately P_i_-depleted waters bordering the North Atlantic subtropical gyre but absent at the maximal rates of P_i_ clearance in the P_i_-depleted gyre (Fig. 9 and Supplementary Table 2).

## Discussion

Accumulation of P_i_ in the periplasm starts with P_i_ diffusion from seawater. The theoretical maximal rate of P_i_ diffusion into the periplasm of a Gram-negative cell depends on a P_i_ diffusion coefficient and the permeable surface area^10,11^. The P_i_ diffusion coefficeint is a seawater trait affecting all cells equally. Cells could, however, control their P_i_-permeable surface area by varying the number of OM porins. *Prochlorococcus* and *Synechococcus* strains respond to P_i_ limitation by the increased expression of P_i_-specific porin genes and the increased abundance of porin proteins^14,40,41^. However, the number of these porins in the OM cannot increase indefinitely. Indeed, the maximal P_i_ clearance rates of oceanic cyanobacterial and SAR11 cells (Fig. 2a) converge towards ~30× below the theoretical maximum. The apparent upper limit to the number of P_i_-specific OM porins is probably a result of the competition for space between porins selective for different small solutes, acquisition of which is essential for a living cell.

In contrast to other solutes, P_i_ somehow accumulates in the periplasm. *Synechococcus* cells rapidly accumulate labile P_i_, which is then steadily internalized and assimilated into macromolecules (Figs. 3 and 4). The semi-labile nature of the accumulated P_i_ (hypotonic removal, Figs. 1c, 3 and 5–7) in conjunction with its resistance to a variety of extracellular treatments (Fig. 6) points to its periplasmic location. Diffusion-driven P_i_ acquisition^11^ can deplete environmental P_i_ concentrations at a constant clearance rate down to 10^−12^ mol l^−1^ (Fig. 2a). This is feasible only if the periplasmic P_i_ concentration of *Synechococcus* cells is <10^−12^ mol l^−1^. Even if we assume that the affinity of the *Synechococcus* PstS subunit for P_i_ is sufficient to bind P_i_ at <10^−12^ mol l^−1^ concentrations (five orders of magnitude below the determined affinity^20^) then every P_i_ molecule that enters and stays in the *Synechococcus* periplasm needs to be instantly bound by a PstS subunit (Fig. 1a†). Sequestration of 6.7 × 10^6^ molecules (Fig. 2b) of labile P_i_ in the periplasm would require an equal number of PstS subunits. The volume of a PstS subunit of 3.5 × 4 × 7 nm dimensions^42^ is ~5.1 × 10^−23^ l, whereas the periplasmic volume of *Synechococcus* is ~2 × 10^−17^ l. This is sufficient to accommodate merely 4 × 10^5^ PstS subunits, or 17× less than the number required. However, even if 4 × 10^5^ PstS subunits cram into the periplasm, there would be no room left for other known periplasmic constituents: substrate binding subunits of at least some of 41 other ABC-type transporters in WH8102^43^, periplasmic enzymes, e.g., alkaline phosphatase^6,44^ and aminopeptidase^45^, and peptidoglycan gel matrix—the major structural component of periplasm^46^. As *Prochlorococcus* cells that have a smaller periplasm^47^ can have as many ABC-type transporters and periplasmic enzymes as WH8102^43^ but accumulate more P_i_ in their buffer (1.2 × 10^7^ molecules^22^), it makes it even less probable that all periplasmic P_i_ is bound by PstS subunits. Finally, up to 20× more P_i_ (e.g., at the 3 h time point in Fig. 3b, d, yellow) remains labile in living *Synechococcus* cells than is incorporated into bacterial macromolecules (which should include PFA-cross-linked PstS subunits, Fig. 1d). As the PstS-mediated P_i_ uptake model (Fig. 1a†) cannot explain the results of our experiments, an alternative explanation is required.

The evident capacity of *Synechococcus* to import the accumulated P_i_ into the cytoplasm (Figs. 3 and 4b) is inconsistent with passive sorption of P_i_ to the outer cell surface^48,49^, because passively adsorbed P_i_ cannot effectively diffuse into the periplasm (Fig. 1a†). Passive sorption of P_i_ by marine bacteria is furthermore questionable, because metabolically inactive but structurally intact bacterioplankton cells (e.g., CCCP-, DBMIB-, DCCD-incapacitated cells) should retain their capacity to adsorb P_i_ passively, but instead, they do not adsorb *P_i_ present in seawater for hours (Supplementary Fig. 5). In contrast, the rapid cessation or slowing down of P_i_ accumulation caused by the specific inhibitors (Fig. 8) and light stimulation of P_i_ accumulation (Fig. 9) indicates that P_i_ accumulation in the periplasm is a metabolically active process.

Light could enhance P_i_ accumulation by SAR11 cells through a proteorhodopsin-generated PMF^50^. Cyanobacteria could use photosynthetically generated PMF for the same purpose. The former energy generator consistently increased P_i_ accumulation by 40%, whereas the latter energy generator showed a higher (≤10×) potential to boost P_i_ accumulation by *Prochlorococcus* cells (Fig. 9). Although such considerable light stimulation is notable, the most unexpected result of the light vs. dark experiments was the lack of light stimulation at the higher rates of P_i_ accumulation (Fig. 9) in the P_i_-depleted waters of the North Atlantic subtropical gyre^22^. Apparently, under severe P_i_ depletion, bacteria do not rely on light as the primary energy source for P_i_ acquisition but use their internal energy sources. P_i_ accumulation seems so important for bacteria that their maximal P_i_ clearance rates (~30× below the theoretical maximal rate of nutrient acquisition by diffusion) are probably constrained by the limited OM surface for incorporating P_i_-specific porins rather than by the availability of cellular energy.

It seems inexplicable that bacteria can accumulate millions of P_i_ molecules (Figs. 2b and 3b, d, and Supplementary Fig. 2b) in their periplasm, while maintaining diffusion even at exceedingly low environmental concentrations. The high percentage of *P_i_ present in the periplasm (Figs. 4–7) and the gradual assimilation of accumulated P_i_ (Figs. 3–4) indicates that the rate of periplasmic P_i_ accumulation by far exceeds the rate of P_i_ import. The parallel import of ^33^P_i_ pulse and ^32^P_i_ chase (Fig. 3) means that the spatial distribution of P_i_ in the periplasm is not ordered. Bacteria do not synthesize polyphosphates in the periplasm but somehow make P_i_ molecules semi-labile: accessible for PstS subunits but prevented from diffusing back into seawater. On the one hand, periplasmic P_i_ needs to remain in ionic form rather than phosphorylate organic molecules, because PstS subunits specifically bind HPO_4_^2−^ and H_2_PO_4_^−^ ions^42^. On the other hand, P_i_ cannot be maintained as free ions because a few free P_i_ ions would make the periplasmic concentration of P_i_ above the environmental concentration and hence reverse diffusion. The nearly instantaneous termination of P_i_ accumulation (Fig. 8a) by the inhibitors CCCP and DBMIB (which dissipate the PMF and thereby halt ATP generation), compared with the delayed reduction of P_i_ accumulation by DCCD (which primarily blocks ATP generation and thus destroys ATP-sustained membrane potential), strongly indicates that P_i_ accumulation is linked to cellular energetics through the PFM rather than being directly driven by ATP through PstSCAB-type transporters (Fig. 1a†). The mutually exclusive conditions (listed above) of PMF-dependent P_i_ accumulation in the periplasm cannot be explained by PstS-mediated P_i_ import (Fig. 1a†). Therefore, an alternative mechanism is needed.

An alternative mechanism of PMF-dependent P_i_ accumulation in the periplasm is a conjecture, because we know little about the organization and functioning of the periplasm^46^ in a living bacterial cell. A cell maintains the PMF by extruding protons (H^+^ ions) across the plasma membrane against the electrochemical gradient using the energy of respiration and photosynthesis^50–52^. Accumulation of H^+^ in the periplasm would make it acidic relative to both the cytoplasm (pH ~ 7.2^53^) and seawater (pH 8.0–8.3^54^) (Fig. 1a‡). However, it is unlikely that many free H^+^ ions would accumulate in the periplasm of marine bacteria, because their OM is permeable to the smallest H^+^ ions. Hence, H^+^ ions should diffuse out into the environment to be neutralized by >100× excess of OH^−^ ions in alkaline (pH 8.0–8.3) seawater^54^, thereby dissipating the membrane potential. To prevent the dissipation of the H^+^-based gradient, H^+^/Na^+^ antiporters may exchange at least some of the periplasmic H^+^ ions for Na^+^ ions^55^. This substitution could preserve the electrical gradient and support ATP production through H^+^-ATP synthases, which can transport Na^+^^56^. Genes for both the H^+^/Na^+^ antiporter and the Na^+^-driven ATP synthase are present in genomes of *Prochlorococcus*^56^ and *Synechococcus* isolates (e.g., NP_875999^56^, WP_010315318, WP_025922676, WP_002805629 and WP_062436653).

The electric potential between the acidic positively charged periplasm and the alkaline seawater would facilitate diffusion, or more precisely mass transfer, of anions with higher negative charge more strongly than anions with lower negative charge. Thereby, mass transfer through anion-selective porins^13^ of HPO_4_^2−^ and PO_4_^3−^ anions (which comprise 29% and 0.01% of the total P_i_ in seawater at pH 8.0, respectively^57,58^) would be particularly favourable. Stronger cationic association of the main seawater anions (e.g., Cl^−^, HSO_4_^−^, HCO_3_^−^ and NO_3_^−^) would also favour mass transfer of free P_i_ anions. Once in the H^+^-enriched periplasm, the HPO_4_^2−^ and PO_4_^3−^ anions would associate with one or two H^+^ and metal cations to reach pH-dependent speciation equilibrium^57,58^. Kinetic stability of neutral P_i_ molecules would explain why disruption of the membrane potential only has a minor immediate effect on the P_i_ already accumulated in the periplasm (Supplementary Fig. 4).

Neutralization of cations with P_i_ anions would reduce the PMF, but that could be restored via continuous extrusion of H^+^ and cations across the plasma membrane to maintain mass transfer of P_i_ anions through porins as long as free P_i_ anions can mass transfer from the environment (Fig. 1a‡). Mass transfer would stop when equilibrium is reached, i.e., all P_i_ anions remaining in seawater are too strongly associated with environmental cations and molecules of water. The strength of their association depends on the total ionic composition of environmental solutions^58^; in seawater, the association of P_i_ anions is apparently weak, because we observed bacterial accumulation of P_i_ anions to continue down to environmental concentrations <10^−12^ mol l^−1^ (Fig. 2) and even <10^−15^ mol l^−1^ (Supplementary Table 4).

Although the periplasm becomes saturated with P_i_ in 1 h (e.g., 4 × 10^6^ P_i_ molecules in 2.1 × 10^−17^ l periplasm equals 0.3 mol l^−1^, Fig. 3b, d), a cell somehow averts precipitation of the P_i_ salts in the periplasm. To prevent formation of insoluble P_i_ salts, a cell would need to minimize concentrations of divalent cations (e.g., Ca^2+^ and Mg^2+^, which associate with P_i_ to form salts with low solubility) in the periplasm, while balancing the concentrations of monovalent cations (e.g., Na^+^ and K^+^) and P_i_ anions to form neutral soluble ion pairs in a way analogous to the common phosphate buffer. Although cation-associated, the H_2_PO_4_^−^ and HPO_4_^2−^ ions remain accessible to PstS-mediated (the PstS subunits have specific affinity to these two anion forms^42^) import, because they remain soluble (Fig. 1a‡). Formation of neutral ion pairs would maintain mass transfer of HPO_4_^2−^ and PO_4_^3−^ into the periplasm, and prevent H^+^ from escaping into seawater. To simplify the explanations we give below, we suggest to term this periplasmic H^+^-driven phosphate-cation association—phosphatation.

Maintaining maximal rates of P_i_ acquisition irrespective of the availability of light energy (Fig. 9) suggests that periplasmic phosphatation is functionally important for marine bacteria as different as cyanobacteria and SAR11 alphaproteobacteria. We propose the following three physiological functions of periplasmic phosphatation: (i) The periplasmic association of P_i_ with chemiosmotic cations accumulates not only P_i_, but also cations. Accumulation of the latter could be viewed as energy conservation, because H^+^/Na^+^ import through the plasma membrane can generate ATP^59,60^. (ii) The high concentration of P_i_ accumulated in the periplasm could also ensure that the PstS transport system is always saturated with P_i_ and operates near its maximal rate. (iii) Periplasmic phosphatation could have an osmotic function. The concentration of P_i_ in the periplasm can increase by 0.3 mol l^−1^ in just 1 h (e.g., 4 × 10^6^ P_i_ molecules in 2.1 × 10^−17^ l periplasm, Fig. 3b, d) and reach >0.5 mol l^−1^ within 3 h. Accumulation of an additional 0.5 mol l^−1^ of P_i_ salt would increase periplasmic osmolarity by ~0.5 osmol l^−1^. Considering that there are other osmotically active molecules in the periplasm, and that the P_i_ salt concentration could raise further the periplasmic osmolarity, this would easily exceed the osmolarity of seawater, i.e., ~1 osmol l^−1^ ^57^. To equilibrate, the resultant osmotic differential water molecules would enter the periplasm increasing its volume and the OM turgor (Fig. 1a‡), explaining how the OM could work as a load-bearing element^61^. Therefore, we predict that periplasmic phosphatation is essential for bacterial osmotic regulation—a hypothesis worth testing experimentally.

There is little doubt that the cationic attraction of P_i_ anions predates life. It was proposed that back in the earliest Archean, positively charged clay particles in oceanic cold alkaline seeps could be viewed as primordial membranes of protocells^62^. Their positive charge would similarly attract P_i_ anions from seawater and this initial enrichment of clay particles with P_i_ might have been inherited by the first living forms, who gradually engaged P_i_ in cellular energetics and later genetics. Thus, periplasmic phosphatation is inherently essential for bacterial cells, because it could conserve energy as well as store and attract the vital P_i_.

## Methods

### Environmental sampling

Data were collected on five oceanographic cruises (Supplementary Table 2) on board the Royal Research Ships *James Clark Ross* (JCR), *James Cook* (JC), *Discovery III* (D) and *Discovery IV* (DY), and the Research Vessel *Maria S. Merian* (MSM) in the North Atlantic during Atlantic Meridional Transect cruises AMT17-D299, AMT20-JC053, AMT22-JC079 and AMT27-DY084 in September–October 2005, 2010, 2012 and 2017, respectively, and during the MSM03 cruise in September–October 2006. At predawn, midday and occasional evening stations seawater samples were collected from 20 m (a representative depth from the surface mixed layer unaffected by the ship’s movement and contamination) using a 20 × 20 l Niskin bottle rosette (Miami, FL, USA) mounted on the stainless steel frame of a conductivity-temperature-depth profiler. Samples were decanted into 10 l polyethylene carboys. Experiments commenced within 1–2 h after sample collection. The concentrations of bioavailable P_i_ were determined using isotope dilution, concentration series bioassays^7,22^ with carrier-free ^32^P-labelled or ^33^P-labelled orthophosphoric acid (Hartmann Analytic GmbH, Braunschweig, Germany). Bioassay experiments were performed in polypropylene, screw cap, 2 ml micro-centrifuge tubes (Starlab, Milton Keynes, UK). Flow-sorting experiments and experiments with inhibitors were performed in Pyrex glass bottles (Fisher Scientific, Loughborough, UK). To minimize artificial contamination (e.g., P_i_, other nutrients, heavy metals), all plastic-ware, glassware and silicone tubing were soaked in 10% HCl and repeatedly rinsed with DW and sampled seawater.

### *Synechococcus* strains and growth conditions

We chose *Synechococcus* sp. WH8102, because its P_i_ acquisition and utilization strategies are known^1,41^. This strain belongs to clade III that is generally considered to be adapted to low-nutrient conditions^63,64^ and abundant in P_i_-depleted Atlantic waters^65^. Strain *Synechococcus* sp. WH8109 is a member of clade II, a clade that dominates tropical and subtropical regions of the world’s ocean^66,67^, tolerates intermediate nutrient-depleted conditions^64,67^ and lives in nutrient-richer upwelling waters^65^. It was reasonable to expect that these strains accumulate P_i_ extracellularly, because they grow better under P_i_-depleted rather than under P_i_-replete conditions^30^. To elucidate whether the P_i_ accumulation was specific to cyanobacterial ecotypes that had evolved to cope with P_i_ deprivation, we also included an opportunistic strain *Synechococcus* sp. WH7803 (clade V) that reaches a maximal growth rate when supplemented with ≥10^−6^ mol l^−1^ P_i_^49^.

Cultures of *Synechococcus* sp. WH8102 and *Synechococcus* sp. WH7803 were axenic. An axenic culture of *Synechococcus* sp. WH8109 strain was established by flow sorting. Live bacterial cells in a mixed culture were stained with 0.1 μg ml^−1^ Hoescht 33342 (final concentration) and *Synechococcus* sp. WH8109 cells were flow cytometrically differentiated from other bacteria by their specific autofluorescence. Using a custom-built MoFlo XDP instrument^68^, WH8109 cells were flow-sorted in batches of 10–200 cells directly into sterile tubes with ASW medium. Tubes were placed into an illuminated growth chamber and incubated until growth became evident by colour.

Axenic cultures of *Synechococcus* sp. strains WH8102, WH8109 and WH7803 were cultivated in an ASW medium^70^ prepared using American Chemical Society grade reagents and supplemented with 8 × 10^−5^ mol l^−1^ K_2_HPO_4_. Semi-continuous cultures were maintained at 23 °C with gentle agitation at 100 r.p.m. on an orbital shaker at a light intensity of ~20 μmol photons m^−2^ s^−1^. Axenic cultures were monitored regularly for contamination by plating ~10^6^ cells on ASW agar plates containing 0.5 g l^−1^ yeast extract and were contaminant free. To maintain trace P_i_ conditions, glassware used for P_i_-free cultivation and experiments was soaked in 10% HCl and thoroughly rinsed with DW.

### Cell enumeration and flow sorting

Cultured *Synechococcus* cells were counted live, unstained, to assess their cell integrity during laboratory experiments (e.g., see Fig. 5c). Oceanic samples were fixed with PFA (1% w/v final concentration), stained with SYBR Green I DNA dye^69^ at 20 °C in the dark for 1 h and flow cytometrically counted and sorted^22,70^ (Supplementary Fig. 1). Bacterioplankton cells flow-sorted during the D299 and JC053 cruises were taxonomically identified using fluorescence in situ hybridization^7,9^. High nucleic acid-containing bacteria with virtually undetectable chlorophyll autofluorescence^71^ were identified as *Prochlorococcus*. Low nucleic acid-containing bacteria were identified as SAR11 alphaproteobacteria. Oceanic *Synechococcus* were identified flow cytometrically based on their specific orange autofluorescence^7^.

### Western blot analyses

Cells from an exponentially growing *Synechococcus* WH8102 culture (OD_750_ = 0.25) were starved for P_i_ to induce PstS synthesis. After 60 h incubation, 800 ml cells were pelleted by centrifugation, washed twice with 20 ml ASW and subjected to 1 min hypotonic shock in 6 ml DW. Following centrifugation, supernatant (6 ml) was collected. The remaining cell pellet was suspended in 6 ml cold DW, supplemented with Protease Inhibitor Cocktail II (Abcam) and the cells were disrupted by two passes through a high-pressure Cell Disruptor (Constant Systems Ltd, Northants, UK). Intact cells were removed from hypotonic DW supernatant and disrupted cells by short centrifugation (5000 × *g*, 10 min, 4 °C). Large membrane debris of disrupted cells were removed using high-speed centrifugation (20,000 × *g*, 60 min, 4 °C) and the remaining cell lysate (6 ml) was collected. Volumes (5, 10 or 20 μl) of DW supernatant and cell lysate were denatured with 1 : 3 (v/v) sample buffer at 90 °C for 5 min and separated by SDS-polyacrylamide gel electrophoresis using 14% acrylamide. A reference gel was stained with Coomassie blue. Western blot immunolabelling used antibodies against the periplasmic phosphate-binding subunit PstS^72^ (1 : 10,000 dilution) and the large Rubisco subunit RbcL (1 : 10,000; AS03 037, Agrisera). For western blotting, proteins were transferred to the polvinylidene difluoride (PVDF) membrane and analysed using standard protocols, following the use of a horseradish peroxidase (HRP)-conjugated goat anti-rabbit IgG HRP-linked secondary antibody (1 : 5000; #7074, Cell Signaling Technology®).

### Pulse-chase experiments

Exponentially growing *Synechococcus* sp. WH8102 cells (0.3–3 × 10^7^ cells ml^−1^) were collected on a 0.2 μm pore size Nuclepore track-etched filter membrane (Whatman International, Ltd, Maidstone, UK) mounted in a filter holder (Swinnex, Millipore, Ireland) and washed with 40 ml P_i_-free ASW (ASW-P_i_) at a flow rate of 2 ml min^−1^ using a syringe pump (KD Scientific, Holliston, MA, USA). The washed cells were pulsed with 10^−8^ mol l^−1^ P_i_ traced with ^33^P_i_ (H_3_^33^PO_4_, specific activity ~111 TBq mmol^−1^; Hartmann Analytic GmbH, Braunschweig, Germany) for 2 h. The pulse was chased with 100× P_i_ concentration (10^−6^ mol l^−1^) traced with carrier-free ^32^P_i_ (H_3_^32^PO_4_, specific activity ~222 TBq mmol^−1^; Hartmann Analytic GmbH, Braunschweig, Germany) and incubated for up to 23 h. Retention of ^33^P_i_ and ^32^P_i_ was determined for live and PFA-fixed cells (Fig. 3). Dissolved tracer was washed from live cells with ASW-P_i_ using a 0.2 μm pore size PVDF membrane spin column (Proteus Mini, Generon, Maidenhead, UK): 0.5 ml cells were placed in a column, spun down at 5000 × *g* for 1 min and washed thrice with 0.1 ml of ASW-P_i_. The radioactivity was determined separately for the cells retained in the spin column insert and for the effluent in the collection tube. To fix cells, 0.5–1.5 ml cells were fixed with 1% PFA, incubated at ambient temperature for 1 h and filtered on 0.2 μm pore size polycarbonate filters. Filters were rinsed twice with 4 ml DW, placed in 20 ml scintillation vials (Meridian, Epsom, UK), mixed with 10 ml scintillation cocktail (Goldstar, Meridian) and radioassayed using a low-activity liquid scintillation analyser (Tri-Carb 3180 TR/SL, PerkinElmer, Beaconsfield, UK) with QuantaSmart™ software. To assess their reproducibility, the pulse-chase experiments were repeated thrice. The results of two experiments are presented in Fig. 3.

### Extracellular P_i_ labelling of *Synechococcus* cells

Cells washed of free P_i_ were re-suspended in 20 ml ASW-P_i_, supplemented with 80–160 Bq ml^−1^ of H_3_^33^PO_4_ or H_3_^32^PO_4_ and incubated in a glass bottle under standard cultivation conditions for 1–3 h. The labelled cells were collected on 0.2 μm pore size Nuclepore filters and effluent was sampled to determine the efficiency of *P_i_ clearance (Supplementary Table 4). Extracellularly *P_i_ pre-labelled *Synechococcus* cells were washed free of residual *P_i_ with 20 ml ASW-P_i_ and re-suspended in solutions appropriate for further experiments.

### Retention of the accumulated P_i_

*P_i_ pre-labelled *Synechococcus* cells were treated with enzymes (Supplementary Table 6) or re-suspended in a range of solutions (Supplementary Tables 5 and 8) using either a 0.2 μm pore size PVDF membrane spin column or a 0.2 μm pore size Nuclepore membrane filter fitted in a 13 mm diameter Swinnex filter holder. For treatments in spin columns, 0.3–0.5 ml treated cells were incubated in a column, spun down at 5000 × *g* for 1 min and washed thrice with 0.1 ml of an appropriate washing solution. The radioactivity was determined separately for the cells retained in the spin column insert and for the effluent in the collection tube. Alternatively, cells were collected on a Nuclepore membrane, washed with 10 ml of a treatment solution at a rate of 0.5–1 ml min^−1^ and the radioactivity determined for the filter membrane only. The activity of the protease Proteinase K in ASW was confirmed using the PDQ protease assay (Athena Environmental Sciences, Inc., Baltimore, MD, USA). To assess their reproducibility, all experiments were repeated independently at least thrice.

### Inhibition experiments

Inhibitors were dissolved in dimethyl sulfoxide (DMSO) and added to samples to a final concentration of 0.05–1 × 10^−4^ mol l^−1^ (Supplementary Table 7) simultaneously with the ^33^P_i_ or ^32^P_i_ tracer. To provide a control, samples were supplemented with the appropriate amount of DMSO. Accumulation of *P_i_ in DMSO-amended samples and control samples without DMSO addition was similar (Shapiro–Wilk normality test passes *P* = 0.932, paired *t*-test *t* = −0.155 with 8 degrees of freedom, two-tailed *P*-value 0.881). Samples were incubated in the light under ambient conditions and sub-samples were withdrawn periodically to determine the total microbial P_i_ accumulation. To assess their reproducibility, all experiments were repeated independently at least thrice.

### Microbeam synchrotron X-ray fluorescence analysis

These measurements were performed at the Diamond Light Source, the UK National Synchrotron Facility. Beamline I18 (Microfocus Spectroscopy) was used to measure the amount of P in cells of *Prochlorococcus* cyanobacteria (range 1–5 × 10^5^ cells) and *Synechococcus* sp. strain WH8102 (0.1–1 × 10^6^ cells) (Supplementary Table 3). Cells were flow-sorted onto 0.2 μm pore size polycarbonate filters (P free) using the custom-built MoFlo XDP instrument^68^. The filters with dried sorted cells were mounted on a custom-made sample holder and the cell-containing filter areas (~1000 × 2000 µm) were probed with a beam spot of 30 × 30 µm^2^ in a helium atmosphere (Supplementary Fig. 3a-c). An approximation of the number of photons on the sample was derived by measuring a thin-film reference material with known mass deposition of a number of metals. This calculated photon flux applied to the cells and accounting for differences in sample matrix, density and volume, leads to an estimated P mass per pixel for the cells. From these values, the total number of P atoms was derived for each cell preparation by integrating signal from all P containing pixels using PyMCA X-ray Fluorescence Toolkit (http://pymca.sourceforge.net) and ImageJ 1.50i (https://imagej.nih.gov/ij/). Using the cultured *Synechococcus* cells as calibrants, sensitivity (≥2 × 10^4^ cells) and linear range (≤7.5 × 10^5^) of the method were determined.

The determined P content was 4.14 ± 1.06 × 10^6^ P atoms per cell for oceanic *Prochlorococcus* equivalent to 1.3–3.1 genomes (accessions: ASM1148v1, ASM1246v1, ASM1264v1, ASM1564v1, ASM1566v1, ASM1858v1, ASM75985v1, ASM792v1 and ASM1146v1). The extracellularly accumulated P_i_ of cells was removed during sorting by the sheath fluid of low osmotic concentration (~0.03 osmol l^−1^), which strips the extracellularly bound P_i_ (Figs. 1, 3, and 5–7). Therefore, only intracellular phosphorus was measured for the sorted cells. In comparison, the P content calculated for an exponentially growing extracellular P_i_-free *Synechococcus* sp. WH8102 cell was 1.9 ± 0.16 × 10^7^ P atoms, which is ~4 times the genomic P content (ASM19597v1).

### Microscopy

Bacterioplankton cells were imaged and their dimensions measured^68^ (Supplementary Fig. 3d-f and Supplementary Table 1). Cells of *Synechococcus* sp. WH8102 were imaged using a LSM780 Confocal Laser Scanning Microscope (Carl Zeiss, Jena, Germany), using 488 nm (Ar) and 594 nm (HeNe) laser lines.

### Statistics and reproducibility

Each experiment was repeated independently at least three times and often more than ten times.

### Reporting summary

Further information on research design is available in the Nature Research Reporting Summary linked to this article.

## Supplementary information

Supplementary Information

Reporting Summary

## Data Availability

The auxiliary data collected on research cruises are archived indefinitely at the British Oceanographic Data Centre (BODC) and are available at www.bodc.ac.uk. The μ-SXRF data are available from Open Science Framework at http://osf.io/6RH2V. All other data supporting the findings of this study are available within the paper and its Supplementary Information files. The source data underlying Figs. 2–4 and 6–9, and Supplementary Figs. 2, 4 and 5 are provided as a Source Data file.

## Source Data

Source Data
