# Supplementary material for: Accumulation of ambient phosphate into the periplasm of marine bacteria is proton motive force dependent
Source: Nat Commun. 2020 May 26;11:2642. doi: 10.1038/s41467-020-16428-w (PMC7250820; doi:10.1038/s41467-020-16428-w)
Supplement: Supplementary file 1 — Supplementary Information [file 41467_2020_16428_MOESM1_ESM.pdf]

Supplementary information for:

Accumulation of ambient phosphate into the periplasm of marine bacteria is proton motive force dependent

Kamennaya et al

The file contains:

Supplementary Figures 1 – 5

Supplementary Tables 1 – 9

Supplementary References 1 – 4

Supplementary Figures

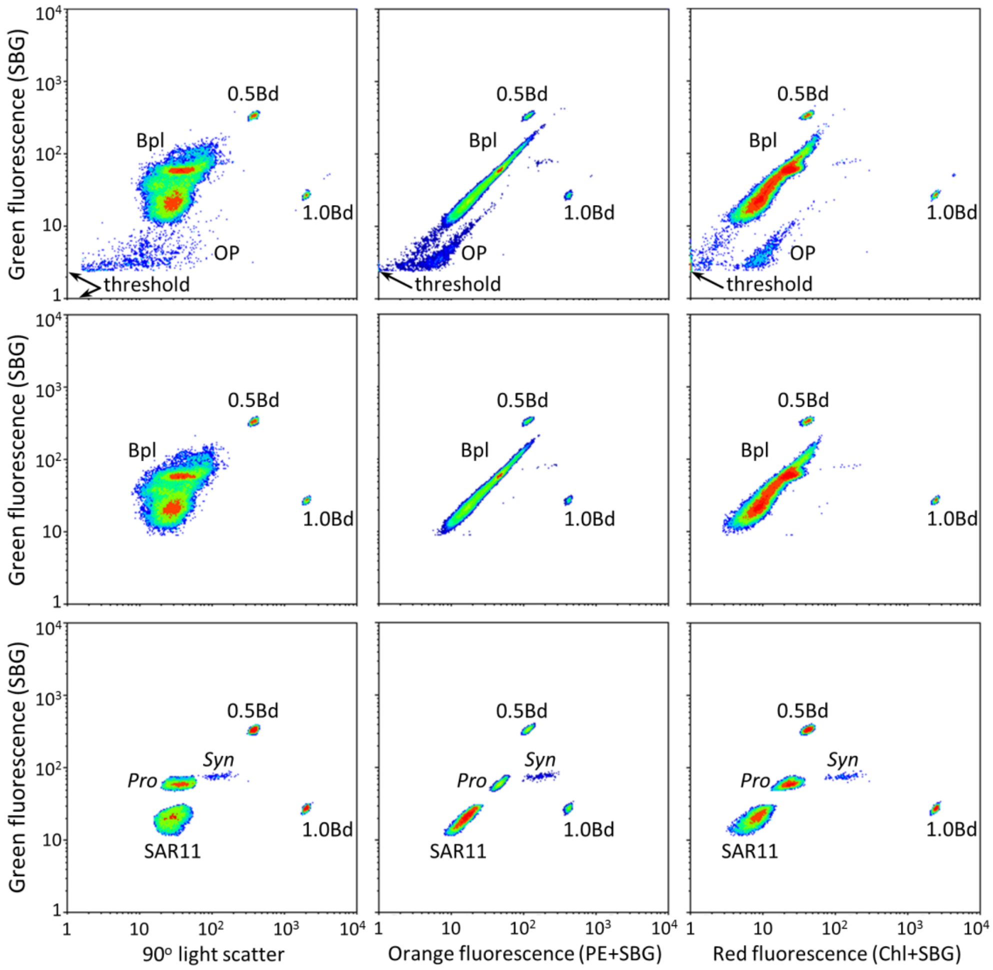

Supplementary Figure 1. Characteristic cytometric signatures of the flow sorted groups of oceanic bacterioplankton.

Density plots of the green (530 ±15 nm) fluorescence as the Y-axis versus the following three X-axis: 90° light scatter (left column), orange (585 ±21 nm) fluorescence (middle column) and red (>650 nm) fluorescence (right column), show the multiplex population of SYBR Green I (SBG) - DNA stained bacterioplankton (Bpl) cells above the set thresholds of the scatter and green fluorescence pointed by the arrows (top row). The scatter and three fluorescence parameters were used to gate the Bpl population (middle row) for sorting from other particles (OP) present in seawater. The same four parameters suffice to discriminate within the Bpl population the three sort-target populations: the dominant population of low nucleic acid-containing, SAR11 cells[1], the second dominant population of *Prochlorococcus* (*Pro*) cells with extra red fluorescence owing to their chlorophyll (Chl) autofluorescence and the minor population of *Synechococcus* (*Syn*) cells with extra orange and red fluorescence owing to their phycoerythrin (PE) and Chl autofluorescence, respectively (bottom row). The SAR11 and *Pro* cells were flow sorted from the gated core of the populations to ensure sorting selectivity. The two reference bead types used are 0.5 µm yellow-green beads (0.5Bd) and 1.0 µm multi-fluorescence beads (1.0Bd).

A total of 2.55 $\times 10^4$ events were recorded for the sample presented, of which 2.08 $\times 10^4$ (81.64%) were gated Bpl cells, 8.14 $\times 10^3$ (31.9%) were gated SAR11 cells, 4.99 $\times 10^3$ (19.6%) were gated *Pro* cells, 76 (0.3%) were gated *Syn* cells, 636 (2.49%) were gated 0.5Bd singlets and 338 (1.33%) were gated 1.0Bd singlets, respectively.

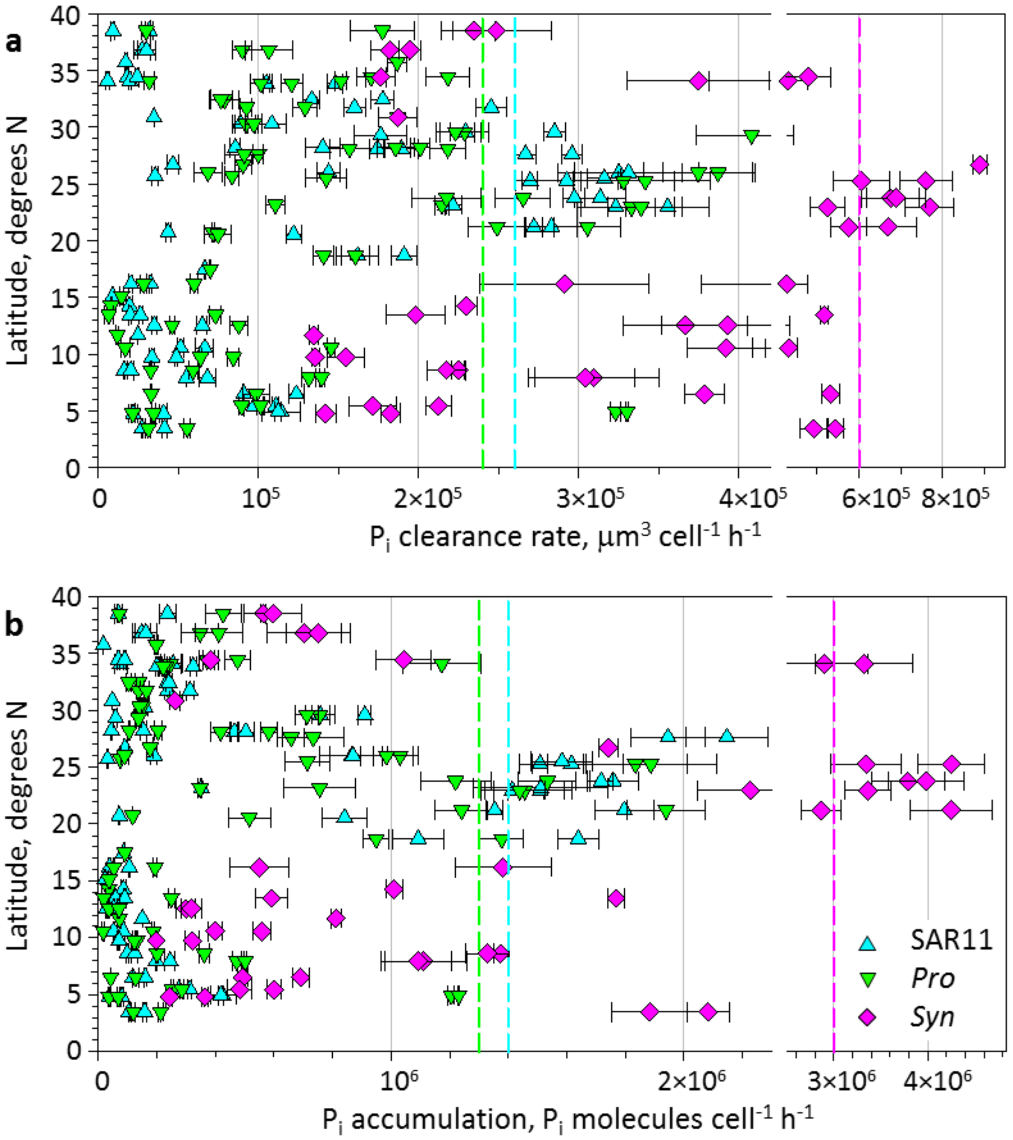

Supplementary Figure 2. Comparison of cellular P_i clearance and accumulation rates of oceanic SAR11, *Prochlorococcus* (*Pro*) and *Synechococcus* (*Syn*) bacteria.

(a) Latitudinal changes in rates of cellular *P_i clearance.
(b) Latitudinal changes in rates of cellular *P_i accumulation.
The plots summarise results of five cruises (Supplementary Table 2). Colour-coded vertical dashed lines indicate the thresholds for selecting the maximal rates presented in Fig. 2. Solid grey lines indicate major X-axis ticks to assist reading and tracing the axis breaks.
Data are presented as mean values of 4 sorting replicates; error bars indicate corresponding SD.

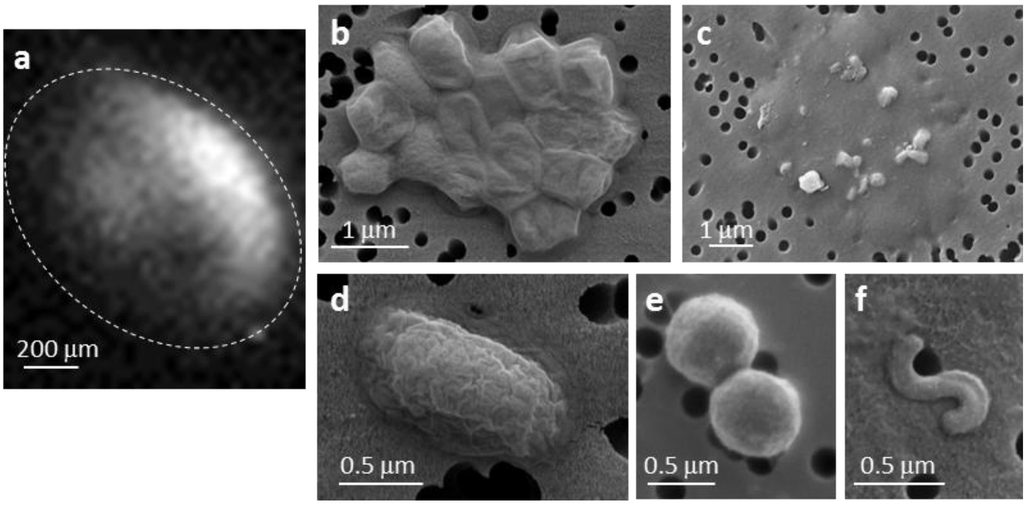

Supplementary Figure 3. Determination of cellular phosphorus content using the μ-SXRF analysis and representative SEM micrographs of flow sorted planktonic bacterial cells.

(a) A phosphorus XRF map of flow sorted *Synechococcus* WH8102 cells.
(b) A SEM micrograph of PFA-fixed *Synechococcus* WH8102 cells flow sorted for the μ-SXRF analysis.
(c) A SEM micrograph of *Synechococcus* cells after the μ-SXRF analysis.
Representative SEM micrographs of flow sorted cells of oceanic **(d)** *Synechococcus* cyanobacteria, **(e)** *Prochlorococcus* cyanobacteria and **(f)** SAR11 alphaproteobacteria. Each experiment was repeated independently 6 or 8 times with similar results. Results of the corresponding μ-SXRF analysis are presented in Supplementary Table 3.

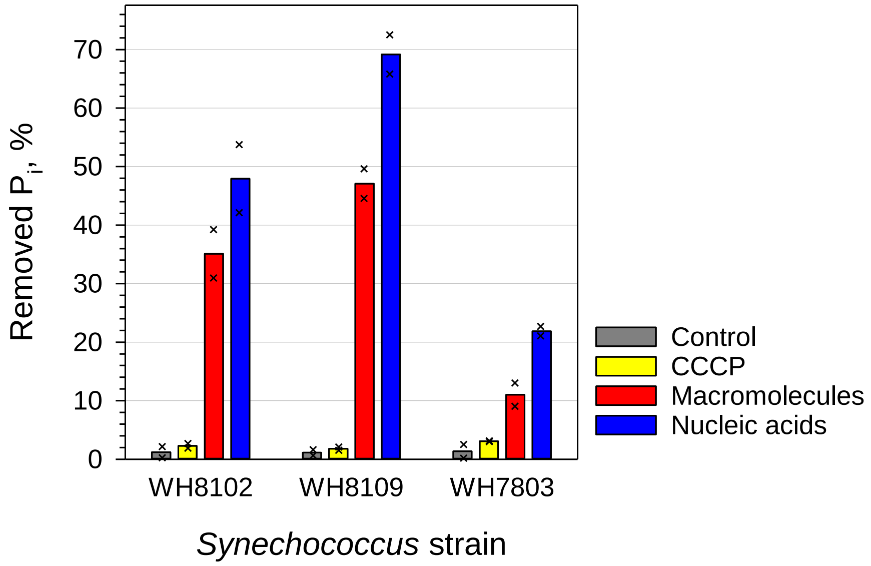

Supplementary Figure 4. Proton motive force independent retention of the accumulated *P_i by pre-labelled cells of *Synechococcus* strains WH8102, WH8109 and WH7803.

The effect of the inhibitor CCCP (Supplementary Table 7) on removal of the accumulated P_i was assessed by comparing the percentages of the $^{33}P_i$ washed by artificial seawater off $^{33}P_i$ pre-labelled live cells before (Control) and after incubation with CCCP for 15 min. These percentages were compared with the percentages of removable P_i from PFA-fixed cellular macromolecules and TCA-precipitated nucleic acids (Fig. 1a, d, e). The plot shows the results of a representative experiment. Data are presented as mean values of technical duplicates indicated as crosses. Horizontal grey lines indicate major ticks of the Y-axis to assist comparing values.

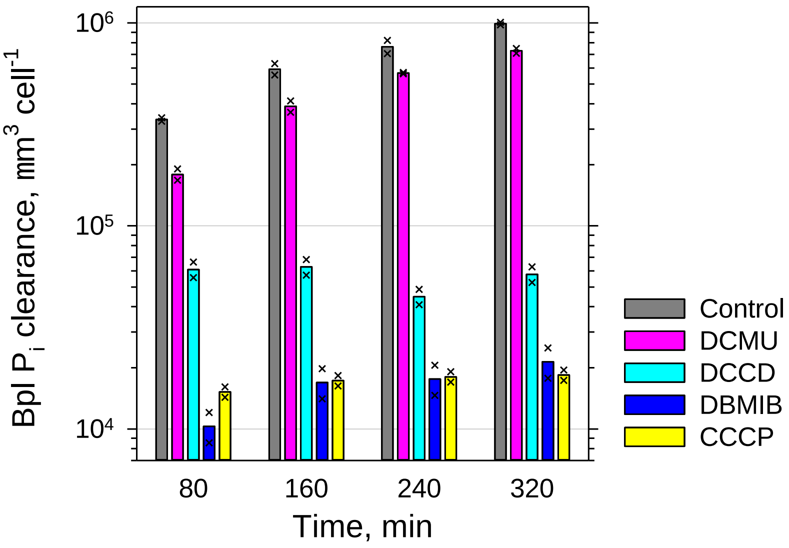

Supplementary Fig. 5. Time course of bacterioplankton (Bpl) *P_i clearance as affected by various metabolic inhibitors (Supplementary Table 7) **compared to the control.**

The plot shows the results of a representative experiment. Data are presented as mean values of technical duplicates indicated as crosses. Horizontal grey lines indicate major ticks of the logarithmic Y-axis to assist comparing values.

Supplementary Tables

Supplementary Table 1

Cellular dimensions, surface area and biovolume of oceanic SAR11, *Prochlorococcus* and *Synechococcus* cells and cultured *Synechococcus* sp. WH8102 cells

	SAR11	*Prochlorococcus*	*Synechococcus*	*Synechococcus* WH8102
Length (μm)	1.14†	0.81†	0.98[2]	1.5
Width (μm)	0.3†	0.81†	0.98[2]	1.0
Depth (μm)	0.3†	0.81†	0.98[2]	1.0
Cell surface (μm^2)	1.08	2.08	3.02	4.71
Biovolume (μm^3)	0.074	0.28	0.49	0.92
Cell surface/volume (μm^{-1})	14.6	7.4	6.1	5.14
n	6	10		10

† The 1.43 coefficient was applied to the SEM-derived dimensions to compensate for dehydration-caused ~30% linear cell shrinkage.

Supplementary Table 2

Sampling dates and geographic positions (Lat °N Long °W) of stations (CTD), at which P_i concentrations, uptake rates and turnover times were determined during five cruises in the North Atlantic Ocean

Date	CTD	Lat °N Long °W	P_i (nmol l^{-1})	P_i rate (nmol l^{-1} h^{-1})	P_i turnover (h^{-1})
28 Oct 2005	8	35°73 29°38	1.56 ±1.01	0.07 ±0.01	22.82 ±12.04
30 Oct 2005	10	30°85 33°1	2.74 ±0.73	0.09 ±0.01	31.68 ±6.47
01 Nov 2005	14	26°7 38°23	5.59 ±0.99	0.51 ±0.03	10.88 ±1.19
02 Nov 2005	17	23°14 36°35	1.80 ±0.86	0.35 ±0.03	5.15 ±1.95
03 Nov 2005	19	20°74 35°12	7.63 ±0.67	0.30 ±0.01	25.54 ±1.06
04 Nov 2005	21	17°48 33°47	2.29 ±0.51	0.11 ±0.01	20.91 ±3.00
05 Nov 2005	23	14°26 31°87	8.02 ±0.67	0.12 ±0.01	68.99 ±2.56
06 Nov 2005	25	11°69 30°61	9.72 ±0.84	0.19 ±0.01	51.45 ±1.84
07 Nov 2005	27	8°61 29°12	4.32 ±0.58	0.22 ±0.01	19.31 ±1.60
07 Nov 2005	28	7°95 28°8	4.54 ±0.30	0.44 ±0.01	10.27 ±0.41
08 Nov 2005	29	6°51 28°11	2.16 ±0.04	0.22 ±0.00	9.80 ±0.12
08 Nov 2005	30	5°46 27°61	5.58 ±0.25	0.44 ±0.01	12.80 ±0.30
08 Nov 2005	31	4°95 27°36	4.19 ±0.47	0.67 ±0.03	6.25 ±0.43
09 Nov 2005	32	3°48 26°66	4.51 ±0.59	0.19 ±0.01	23.86 ±1.43
28 Sep 2006	16	38°48 30°00	2.52 ±0.64	0.12 ±0.01	20.60 ±3.66
29 Sep 2006	17	36°78 30°00	2.33 ±1.00	0.15 ±0.15	15.75 ±4.84
30 Sep 2006	18	34°07 30°00	2.04 ±0.85	0.10 ±0.10	19.80 ±6.10
01 Oct 2006	19	34°41 28°48	2.25 ±0.15	0.10 ±0.10	22.47 ±1.08
21 Sep 2010	21	33°84 30°20	2.40 ±0.37	0.45 ±0.05	5.29 ±0.63
22 Sep 2010	23	32°43 31°80	1.77 ±0.09	0.19 ±0.01	9.24 ±0.39
23 Sep 2010	24	31°73 32°56	1.55 ±0.33	0.19 ±0.02	8.28 ±1.57
23 Sep 2010	26	30°29 34°18	1.00 ±0.18	0.20 ±0.01	5.02 ±0.87
24 Sep 2010	27	29°58 34°61	2.47 ±0.09	0.71 ±0.02	3.50 ±0.09
25 Sep 2010	29	28°11 36°51	3.82 ±0.38	0.59 ±0.04	6.46 ±0.48
25 Sep 2010	33	25°98 38°78	3.23 ±0.16	1.21 ±0.04	2.67 ±0.10
26 Sep 2010	34	25°27 39°53	5.31 ±0.41	2.10 ±0.12	2.53 ±0.12
26 Sep 2010	36	23°77 41°11	3.77 ±0.29	1.59 ±0.07	2.36 ±0.15
26 Sep 2010	37	22°96 40°53	5.14 ±0.38	1.64 ±0.08	3.13 ±0.17
27 Sep 2010	39	21°21 39°29	5.34 ±0.44	1.59 ±0.09	3.37 ±1.97
28 Sep 2010	42	18°69 37°52	6.02 ±0.46	1.25 ±0.07	4.82 ±0.25
29 Sep 2010	45	16°19 35°80	5.32 ±0.61	0.09 ±0.0	56.12 ±0.76
30 Sep 2010	48	13°47 33°95	10.57 ±1.60	0.08 ±0.01	138.11 ±8.62
30 Sep 2010	49	12°55 33°33	3.43 ±0.25	0.06 ±0.00	56.87 ±3.30
31 Sep 2010	51	10°57 32°00	3.98 ±0.68	0.11 ±0.01	37.70 ±4.96
31 Sep 2010	52	09°75 31°46	4.12 ±0.43	0.31 ±0.02	13.40 ±1.02
2 Oct 2010	57	04°80 28°16	9.35 ±1.73	0.09 ±0.01	105.81 ±13.98
21 Oct 2012	19	27°61 36°35	2.69 ±0.19	1.05 ±0.11	2.55 ±0.18
22 Oct 2012	21	25°50 38°98	2.50 ±0.14	0.85 ±0.05	2.96 ±0.17
23 Oct 2012	23	23°16 40°60	2.16 ±0.38	0.71 ±0.11	3.03 ±0.53
24 Oct 2012	24	20°57 38°59	2.30 ±0.20	0.47 ±0.04	4.86 ±0.41
26 Oct 2012	26	15°13 34°45	2.55 ±0.01	0.05 ±0.00	48.25 ±0.18
30 Sep 2017	10	35°19 26°18	1.52 ±0.43	0.19 ±0.02	7.93 ±2.01
1 Oct 2017	13	32°54 26°53	0.32 ±0.09	0.78 ±0.02	1.69 ±0.11
1 Oct 2017	14	31°46 27°11	0.85 ±0.17	0.50 ±0.03	1.80 ±0.35
2 Oct 2017	15	29°19 27°47	0.38 ±0.08	0.08 ±0.00	4.72 ±0.98
2 Oct 2017	16	28°12 28°04	1.47 ±0.32	0.24 ±0.02	6.19 ±1.20
3 Oct 2017	18	26°01 35°30	1.26 ±0.26	0.20 ±0.02	6.26 ±1.20
3 Oct 2017	19	25°43 28°40	1.98 ±0.16	0.09 ±0.00	21.07 ±1.40
4 Oct 2017	19	25°43 28°40	0.43 ±0.28	0.20 ±0.02	2.20 ±1.43
4 Oct 2017	20	23°22 29°12	9.23 ±2.48	0.01 ±0.00	734.30 ±49.00

The mean values ± SEM derived from the linear regression slope of the bioassay experiments, each comprised of 24 individual measurements[3].

Supplementary Table 3

Cellular phosphorus (P) contents of quantitatively flow sorted oceanic LNA, *Prochlorococcus* and cultured *Synechococcus* cells

	LNA	*Prochlorococcus*	*Synechococcus* sp. WH8102
P atoms cell^{-1}	2.68×10^6	4.14×10^6	1.93×10^7
SD	1.3×10^6	1.06×10^6	1.57×10^6
n	6	8	6

LNA, low nucleic acid bacterioplankton cells are dominated by SAR11[1]; SD, standard deviation; n, number of replicates.

Supplementary Table 4

The percentage (%) of $^{32}P_i$ acquired from ^{32}P-spiked ASW-P_i by cultured *Synechococcus*

Strain	Cell concentration (cells ml^{-1})	Time (h)	%†
WH7803	1.5×10^7	2	≥99
	5×10^7	12	≥99
WH8109	4×10^7	2	≥99
	5×10^7	12	≥99
WH8102	4×10^7	2.5	97
	1×10^6 - 1×10^7	5	≥99
	1×10^6 - 1×10^7	5	≥99
	5×10^7	12	≥99

† The sensitivity of ^{32}P radiotracer method limits the measurement precision to 99%.

Supplementary Table 5

Chemical solutions used to assess the stability of the accumulated P_i

Solution	pH	Treatment duration (h)	Mechanism of P_i removal
ASW -P_i	8.0	0.03, 0.25, ≤48	Depletion
ASW -P_i	6.0	0.03, 0.25	Protonation
ASW -P_i	10.0	0.03, 0.25	De-protonation
ASW -P_i -Ca	8.0	0.03, 0.25	Destabilization of Ca-bound complexes
0.45 mol l^{-1} NaCl	8.0	0.03, 0.25	Destabilization of metal-bound
0.45 mol l^{-1} NaCl + 0.3 mol l^{-1} EDTA	8.0	0.03, 0.25	complexes
Pluronic® F-68 0.01%, 0.04%	8.0	0.25, 12, 20	Cell membrane destabilization
Triton X-100 0.01, 0.02, 0.04 %	8.0	0.25, 12, 20	Cell membrane destabilization

Supplementary Table 6

Enzymatic treatments used to identify the location of the accumulated P_i

Enzyme (buffer)	Concentrations	Treatment duration (h)	Enzymatic activity	Manufacturer
Proteinase K	1, 10 μg ml^{-1}	0.25, 0.9, 1, 20	Protein degradation	Promega
Egg white lysozyme	1, 10, 100 mg ml^{-1}	0.25, 0.9, 12, 20	Electrostatic interaction with LPS[4]	Amresco
Alkaline phosphatase	1.25, 25, 50, 100 u ml^{-1}	0.5, 0.9, 1, 2.5, 12, 15, 20	De-phosphorylation	New England Biolabs

Supplementary Table 7

Inhibitors used to study P_i accumulation

Treatment (buffer)	Concentrations ($\times 10^{-5}$ mol l^{-1})	Treatment duration (h)	Inhibitory effect
3-(3,4-dichloro-phenyl)-1,1-dimethylurea (DCMU)	0.5, 1, 10	0.2, 0.5, 0.7, 1, 1.3, 2, 2.7, 4, 5.3, 6.7, 18	Partially inhibits light-dependent membrane polarization, reduces ATP generation
2,5-dibromo-3-methyl-6-isopropylbenzo-quinone (DBMIB)	1, 10	0.2, 0.3, 0.5, 0.7, 1, 1.3, 2, 2.67, 4, 5.3, 6.67	Inhibits electron transport-dependent membrane polarization, hence inhibits ATP generation
N,N'-dicyclohexyl-carbodiimide (DCCD)	1, 10	0.3, 0.5, 0.7, 1, 1.3, 2, 2.6, 4, 5.3, 6.6	Inhibits ATP generation
Carbonyl cyanide m-chloro-phenyl-hydrazone (CCCP)	1, 10	0.3, 1, 2, 2.5, 12, 15, 18	Depolarizes membranes, hence inhibits ATP generation
Monensin	10	0.5, 1, 2, 4	Ionophore that transports monovalent cations (i. e. Na^+, K^+) between intracellular and extracellular spaces in electroneutral mode to equilibrate concentrations of the ions
Valinomycin	0.5, 2.5, 10	0.5, 1, 1.5, 2, 4	K^+-specific ionophore
A23187	10	0.5, 1, 2, 4	Divalent cations (i. e. Mn^{2+}, Ca^{2+}, Mg^{2+})-specific ionophore

Supplementary Table 8

Hypotonic chemical solutions used to assess the stability of the accumulated P_i as compared to ASW

Solution	pH	Treatment duration (h)	Osmotic concentration (osmol l^{-1})	P_i removal
DW	7.0	0.03, 0.5, 12	0	+
1:100 ASW:DW	8.0	0.03	0.01	+
1:40 ASW:DW	8.0	0.03	0.025	+
1:10 ASW:DW	8.0	0.03	0.1	+
1:10 ASW:DW + P_i 0.05 mol l^{-1}	7.5	0.67	0.15	+
Phosphate buffered saline (PBS)	7.5	2, 16	0.31	+
ASW	8.0	variable $\leq$48	1.0	-

Supplementary Table 9

Comparison of P_i clearance rates of oceanic SAR11, *Prochlorococcus* (*Pro*) and *Synechococcus* (*Syn*) cells in the light versus dark

	n	Z-Statistic	Statistically significant difference (*P*)
SAR11	28	4.031	<0.001
Pro	32	4.020	<0.001
Syn	19	2.133	0.032

Results of Wilcoxon's signed rank test of the data shown on the Fig. 9.

Supplementary References

1 Gomez-Pereira, P. R. *et al.* Comparable light stimulation of organic nutrient uptake by SAR11 and *Prochlorococcus* in the North Atlantic subtropical gyre. *ISME J* **7**, 603-614, doi:10.1038/ismej.2012.126 (2013).

2 Zubkov, M. V., Sleigh, M. A., Burkill, P. H. & Leakey, R. J. G. Picoplankton community structure on the Atlantic Meridional Transect: a comparison between seasons. *Prog Oceanogr* **45**, 369-386, doi:10.1016/S0079-6611(00)00008-2 (2000).

3 Zubkov, M. V., Martin, A. P., Hartmann, M., Grob, C. & Scanlan, D. J. Dominant oceanic bacteria secure phosphate using a large extracellular buffer. *Nat Commun* **6**, 7878, doi:10.1038/ncomms8878 (2015).

4 Masschalck, B. & Michiels, C. W. Antimicrobial properties of lysozyme in relation to foodborne vegetative bacteria. *Crit Rev Microbiol* **29**, 191-214, doi:10.1080/713610448 (2003).
